# Microbiota-indole 3-propionic acid-brain axis mediates abnormal synaptic pruning of hippocampal microglia and susceptibility to ASD in IUGR offspring

**DOI:** 10.1186/s40168-023-01656-1

**Published:** 2023-11-07

**Authors:** Tingting Wang, Beidi Chen, Mingcui Luo, Lulu Xie, Mengxi Lu, Xiaoqian Lu, Shuai Zhang, Liyi Wei, Xinli Zhou, Baozhen Yao, Hui Wang, Dan Xu

**Affiliations:** 1 Department of Obstetrics, Zhongnan Hospital of Wuhan University, School of Pharmaceutical Sciences, Wuhan University, Wuhan, 430071 China; 2 Department of Rheumatology and Immunology, Peking University Third Hospital, Beijing, 100191 China; 3 Department of Pediatrics, Renmin Hospital of Wuhan University, Wuhan, 430071 China; 4 Department of Pharmacology, Taikang Medical School (School of Basic Medical Sciences), Wuhan University, Wuhan, 430071 China; 5 Hubei Provincial Key Laboratory of Developmentally Originated Disease, Wuhan, 430071 China

**Keywords:** Intrauterine growth restriction, Autism spectrum disorder, Gut microbiota, Indole 3-propionic acid, Microglia synaptic pruning

## Abstract

**Background:**

Autism spectrum disorder (ASD) has been associated with intrauterine growth restriction (IUGR), but the underlying mechanisms are unclear.

**Results:**

We found that the IUGR rat model induced by prenatal caffeine exposure (PCE) showed ASD-like symptoms, accompanied by altered gut microbiota and reduced production of indole 3-propionic acid (IPA), a microbiota-specific metabolite and a ligand of aryl hydrocarbon receptor (AHR). IUGR children also had a reduced serum IPA level consistent with the animal model. We demonstrated that the dysregulated IPA/AHR/NF-κB signaling caused by disturbed gut microbiota mediated the hippocampal microglia hyperactivation and neuronal synapse over-pruning in the PCE-induced IUGR rats. Moreover, postnatal IPA supplementation restored the ASD-like symptoms and the underlying hippocampal lesions in the IUGR rats.

**Conclusions:**

This study suggests that the microbiota-IPA-brain axis regulates ASD susceptibility in PCE-induced IUGR offspring, and supplementation of microbiota-derived IPA might be a promising interventional strategy for ASD with a fetal origin.

Video Abstract

**Supplementary Information:**

The online version contains supplementary material available at 10.1186/s40168-023-01656-1.

## Background

Autism spectrum disorder (ASD) is a kind of developmental mental disorder that usually occurs in childhood and is characterized by social communication deficits, restricted interests, and stereotyped behaviors. The increasing incidence of ASD worldwide has become a public health problem of global concern [1–3]. The “developmental origins of health and disease (DOHaD)” theory has received extensive attention in recent years, focusing on the fetal origin of human diseases [4]. Intrauterine growth restriction (IUGR) is an adverse event in the uterus that causes an infant to be born with a birth weight two standard deviations below the mean weight for age or below the 10th percentile for age [5]. IUGR is a common obstetric disease and the leading cause of premature birth, respiratory distress, and even death [6, 7]. In the past two decades, the incidence of IUGR has also increased rapidly, with an incidence rate up to nearly 30% in developing countries, much higher than that in developed countries [8, 9]. Epidemiological survey showed that children with IUGR would be susceptible to various chronic diseases when they become an adult, which includes ASD [10]. These findings suggest that ASD has a fetal developmental origin. However, the underlying mechanisms of ASD with a fetal origin remain unclear.

Caffeine is a xanthine alkaloid that widely exists in coffee, tea, energy drinks, food, and painkillers [11]. Prenatal caffeine exposure (PCE) is very common, with approximately 60 to 75% of pregnant women drinking caffeinated beverages [12]. In addition, there is a large amount of caffeine residue in the environment, and pregnant women can be unconsciously exposed to them [13]. Over the past two decades, there has been a notable rise in the levels of caffeine and its derivatives in water bodies in our surroundings [14]. Additionally, prolonged exposure to contaminated water can result in the transfer and accumulation of caffeine in organisms through the food chain, ultimately leading to the bioaccumulation of caffeine residues in human bodies [15]. Epidemiological survey showed that caffeine consumption during pregnancy was associated with an increased risk of IUGR, and this association continued throughout pregnancy [16]. Studies based on neonatal anthropometry have found that caffeine consumption during pregnancy, even at levels much lower than the recommended 200 mg per day, is associated with decreased fetal growth [17]. Recently, PCE was reported to be closely associated with low birth weight, the primary manifestation of IUGR [18]. PCE can also affect children’s brain development and cognition [19, 20]. Our previous study has confirmed that PCE can cause IUGR and impaired neural development in offspring rats [21, 22]. However, whether the PCE-induced IUGR offspring are susceptible to ASD is still unknown.

The composition and function of gut microbiota, the microbial population colonizing the host gut, are closely associated with the development and function of the host nervous system [23, 24]. Patients with neuropsychiatric diseases, including ASD, have been reported to have altered gut microbiota compared to healthy people [25]. An increased gut *Bacteroides* residency was demonstrated to promote disease progression in an ASD mouse model by inhibiting intestinal amino acid transport and reducing serum glutamine levels [26]. In addition, transplanting the fecal microbiota from healthy people to ASD patients can relieve their symptoms, such as lethargy, stereotypy, and inappropriate speech [27]. These results suggest that abnormal gut microbiota is implicated in ASD pathogenesis. Furthermore, prenatal antibiotic exposure could cause compositional changes in infant gut microbiota as well as an increased risk of ASD [28, 29], indicating that the gut microbiota-associated ASD susceptibility can be traced back to the intrauterine period.

Gut microbiota can regulate the development and function of host nervous system through its metabolites [30–32]. Indole 3-propionic acid (IPA) is one of the main tryptophan metabolites of gut microbiota, which cannot be produced by human cells [33]. IPA is also an endogenous ligand of the aryl hydrocarbon receptor (AHR) capable of crossing the blood-brain barrier. Transfer of fecal microbiota from ASD children to germ-free mice led to changes in microbiota-related tryptophan metabolism and ASD-like symptoms [34], suggesting an association between the tryptophan metabolism by gut microbiota, like IPA production, and ASD. ASD patients are known to have structural changes in the hippocampus, accompanied by hyperactivation of hippocampal microglia and abnormal synaptic pruning of neurons [35]. A mechanistic study showed that microglia hyperactivation could be a result of AHR deletion [36]. In addition, our previous metabolomic study showed that the serum IPA level was markedly reduced in PCE-induced IUGR rats [37]. Thus, the downregulation of IPA/AHR signaling mediated by abnormal gut microbiota might be an essential player in the ASD development of PCE-induced IUGR offspring.

In this study, we verified the ASD susceptibility in young IUGR offspring utilizing the established PCE-induced IUGR rat model. Then, we explored the hippocampus-related cellular and molecular mechanisms underlying the ASD-like symptoms in the IUGR rats. Finally, combining the serum data from IUGR children and the interventional experiments in rats, we decipher the role of the gut microbiota-metabolite-brain axis in the pathogenesis of ASD with a fetal origin. The results of our study laid a theoretical and experimental foundation for establishing preventional and therapeutic strategies for ASD patients.

## Results

### PCE-induced IUGR rats develop ASD-like symptoms with microglia activation and neuronal synapse over-pruning in hippocampus

**Fig. 1:**
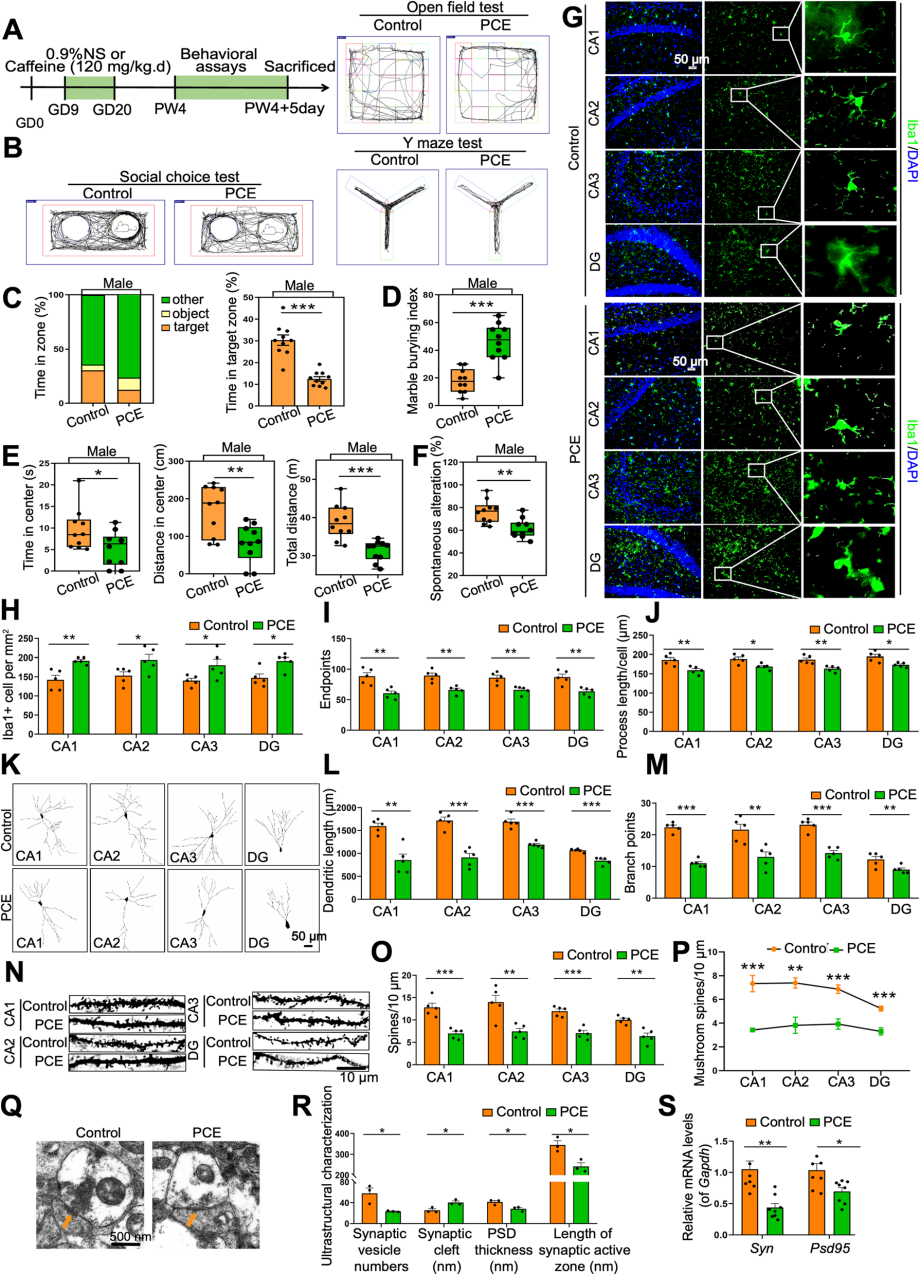
PCE-induced IUGR rats develop ASD-like symptoms with microglia activation and neuronal synapse over-pruning in hippocampus. **A** Schematic of the experimental design. PW, postnatal week. GD, gestational day. **B** An illustrative example of travel pathways of rats in social choice test, open field test, and Y maze test. **C** Time of rats spent in each zone in social choice test. **D** Marble burying index in marble burying test. **E** Time spent in the center, distance traveled in the center, and total distance traveled in open field test. **F** Spontaneous alteration rate in Y maze test. **G** Images under fluorescence microscopy showing different regions in hippocampus. Iba1 staining (green) and nuclear staining (DIPA, blue). Scale bar = 50 μm. **H**–**J** Number of Iba1^+^ cells, number of endpoints per microglia, and process length. **K**–**P** Golgi staining. **K** Representative reconstruction of the hippocampal neurons. Scale bar = 50 μm. **L** Dendritic length of hippocampal neurons. **M** Number of branch points in hippocampal neurons. **N** Representative images of dendritic segments. Scale bar =10 μm. **O** Total spine density in hippocampal neurons. **P** Mushroom spine density in hippocampal neurons. **Q** Ultrastructure of neuronal synapses. Scale bar = 500 nm. **R** Synaptic vesicle numbers, synaptic cleft, postsynaptic density (PSD) thickness, and length of synaptic active zone. **S** mRNA levels of *Syn* and *Psd95*. Dots in panels represent individual samples. Data are presented as mean ± SEM. ^*^P < 0.05, ^**^P < 0.01, and ^***^P < 0.001

Based on epidemiological investigations and our previous studies [16, 18, 21, 22], we established a stable IUGR model by gavaging pregnant rats with caffeine at a dose that is accessible to pregnant women in daily life according to the human-rat dose conversion formula (*P* < 0.001, Fig. S1A-D). We first assessed the ASD-like symptoms of PCE-induced IUGR rats (hereafter referred to as the IUGR rats) and non-IUGR rats (hereafter referred to as the control rats) at 4 weeks of age (postnatal week 4, PW4) (Fig. 1A). In the social choice test, the IUGR male rats had less interaction with the rat-targeted area and spent more time exploring other areas compared to the control rats (*P* < 0.001, Fig. 1B, C). In the marble burying test, more marbles were buried by the IUGR male rats (*P* < 0.001, Fig. 1D). In the open field test, the total distance moved in all regions (*P* < 0.001) as well as the distance moved (*P* = 0.001) and the time taken (*P* = 0.040) in the central region were significantly reduced in the IUGR male rats (Fig. 1B, E). In the Y maze test, the spontaneous alteration rate of IUGR male rats was decreased (*P* = 0.002, Fig. 1B, F). These results suggested that PCE-induced IUGR male rats could spontaneously develop ASD-like symptoms, such as stereotyped repetitive behaviors, social communication deficits, reduced free exploration, and reduced spatial memory. We also observed the behavior of PCE-induced IUGR female rats and found that they only showed decreased ability of free exploration and spatial memory (Fig. S1E-I). Additionally, males are four times more likely to develop ASD than females [38]. Thus, we focused on male rats instead of female rats in the following experiments.

The hippocampal lesions have been associated with the social memory deficits of ASD [39–41]. Next, we examined the hippocampus changes of PCE-induced male IUGR rats at PW4. Compared with the control rats, the number of microglia in the hippocampal CA1, CA2, CA3, and DG regions of the IUGR rats significantly increased, while the endpoints and process length of microglia decreased (*P* < 0.05 or *P* < 0.01, Fig. 1G–J). These phenomena indicated the activation of microglia in IUGR rats. Synapses are essential hubs for information transmission between neurons. Activated microglia can affect brain function by pruning neuron synapses and regulating synaptic plasticity [42]. According to the Golgi staining, dendritic length, branch points, and dendritic complexity of neurons in the hippocampal CA1, CA2, CA3, and DG regions were all decreased in IUGR rats (*P* < 0.01 or *P* < 0.001, Fig. 1K–M), suggesting synapse over-pruning in the hippocampal neurons. Over-pruning of synapses can lead to synapse impairment, manifested by decreased synapse number, altered synaptic structure, and downregulated excitatory synaptic indicators. Both the total spine density and the mushroom spine density of neurons in the hippocampal CA1, CA2, CA3, and DG regions in IUGR rats were decreased (*P* < 0.01 or *P* < 0.001, Fig. 1N–P), indicating a decreased synapse number in the hippocampus of IUGR rats. We then focused on the ultrastructural changes of synapses in the hippocampal CA2 region, the region responsible for social memory, by transmission electron microscopy (TEM). We found decreased synaptic vesicle numbers, widened synaptic cleft, decreased postsynaptic density (PSD) thickness, and reduced length of synaptic active zone in the hippocampal CA2 region in IUGR rats (*P* < 0.05, Fig. 1Q, R). Additionally, the expression levels of excitatory synaptic markers *Syn* and *Psd95* in the hippocampus of IUGR rats were also significantly decreased (*P* < 0.05 or *P* < 0.01, Fig. 1S).

Taken together, compared with control rats, PCE-induced male IUGR rats exhibited ASD-like symptoms at PW4, accompanied by microglia hyperactivation as well as subsequent synapse over-pruning and impairment in the hippocampus.

### Dysregulated AHR/NF-κB signaling mediates hippocampal pathology and ASD susceptibility in PCE-induced IUGR rats

**Fig. 2:**
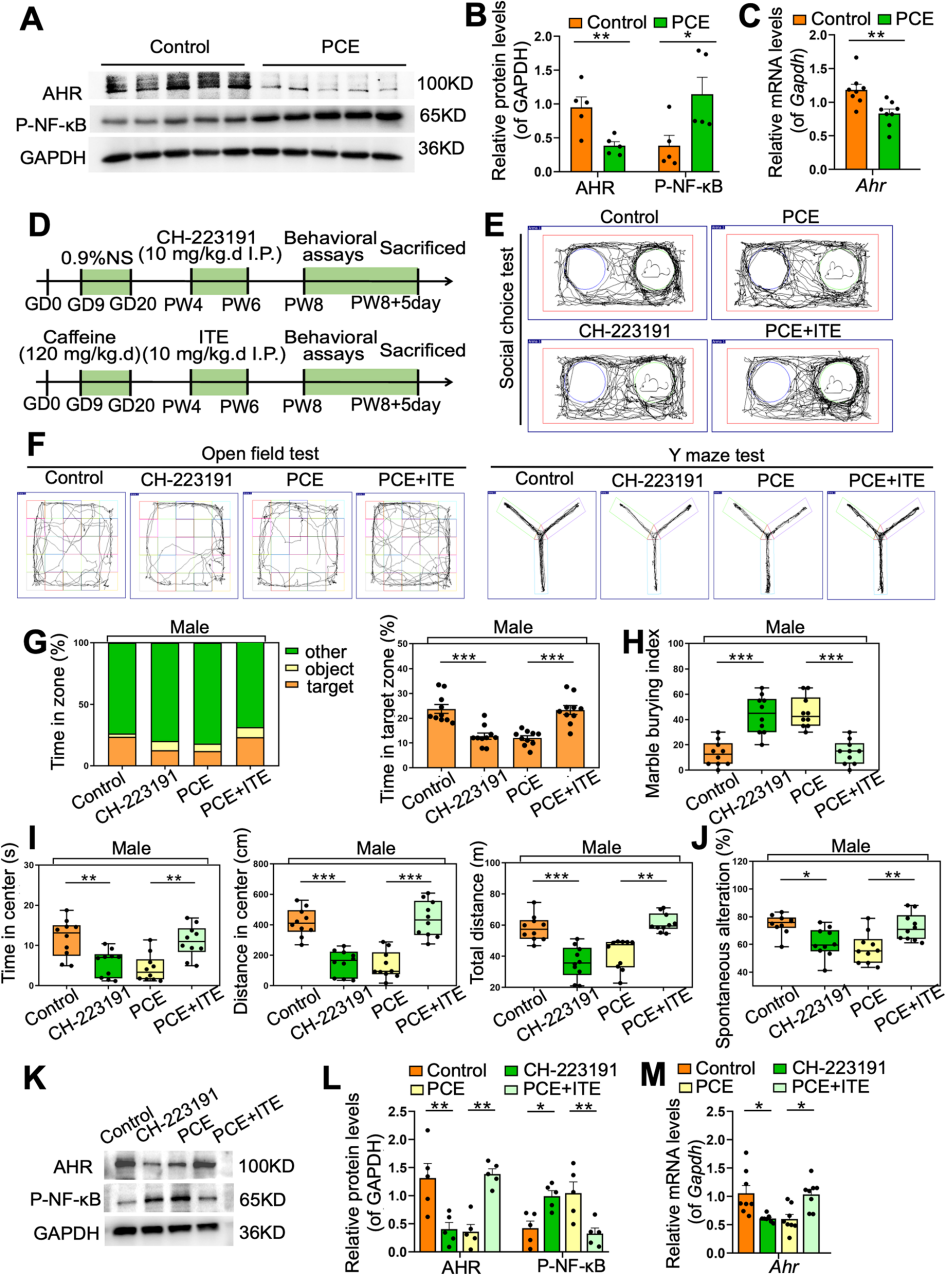
Dysregulated AHR/NF-κB signaling mediates ASD susceptibility in PCE-induced IUGR rats. **A**, **B**, **K**, **L** Protein levels of AHR and P-NF-κB. **C**, **M** mRNA level of *Ahr*. **D** Schematic of the experimental design in vivo. **E**, **F** An illustrative example of travel pathways of rats in social choice test, open field test, and Y maze test. **G** Time of rats spent in each zone in social choice test. **H** Marble burying index in marble burying test. **I** Time spent in the center, distance traveled in the center, and total distance traveled in open field test. **J** Spontaneous alteration rate in Y maze test. Dots in panels represent individual samples. Data are presented as mean ± SEM. ^*^P < 0.05, ^**^P < 0.01, and ^***^P < 0.001

Next, we explored the molecular mechanism of microglia hyperactivation in the hippocampus of PCE-induced male IUGR rats. Increased activity of nuclear factor kappa-B (NF-κB) has been associated with ASD pathogenesis [43, 44]. We also analyzed the published transcriptomic data of hippocampal tissue in an ASD mouse model and identified 14 significantly changed signal pathways, among which was the upregulated NF-κB signal pathway (Fig. S2A, S2B). It was reported that the level of phosphorylated-NF-κB (P-NF-κB) increased after the AHR knockout in macrophages [45], suggesting an interaction between NF-κB and AHR. Notably, AHR was downregulated while P-NF-κB was upregulated in the hippocampus of the IUGR rats compared to the control rats (*P* < 0.05 or *P* < 0.01, Fig. 2A–C). Therefore, we hypothesized that AHR downregulation and subsequent NF-κB activation in the hippocampus might mediate the ASD susceptibility in PCE-induced male IUGR rats.

**Fig. 3:**
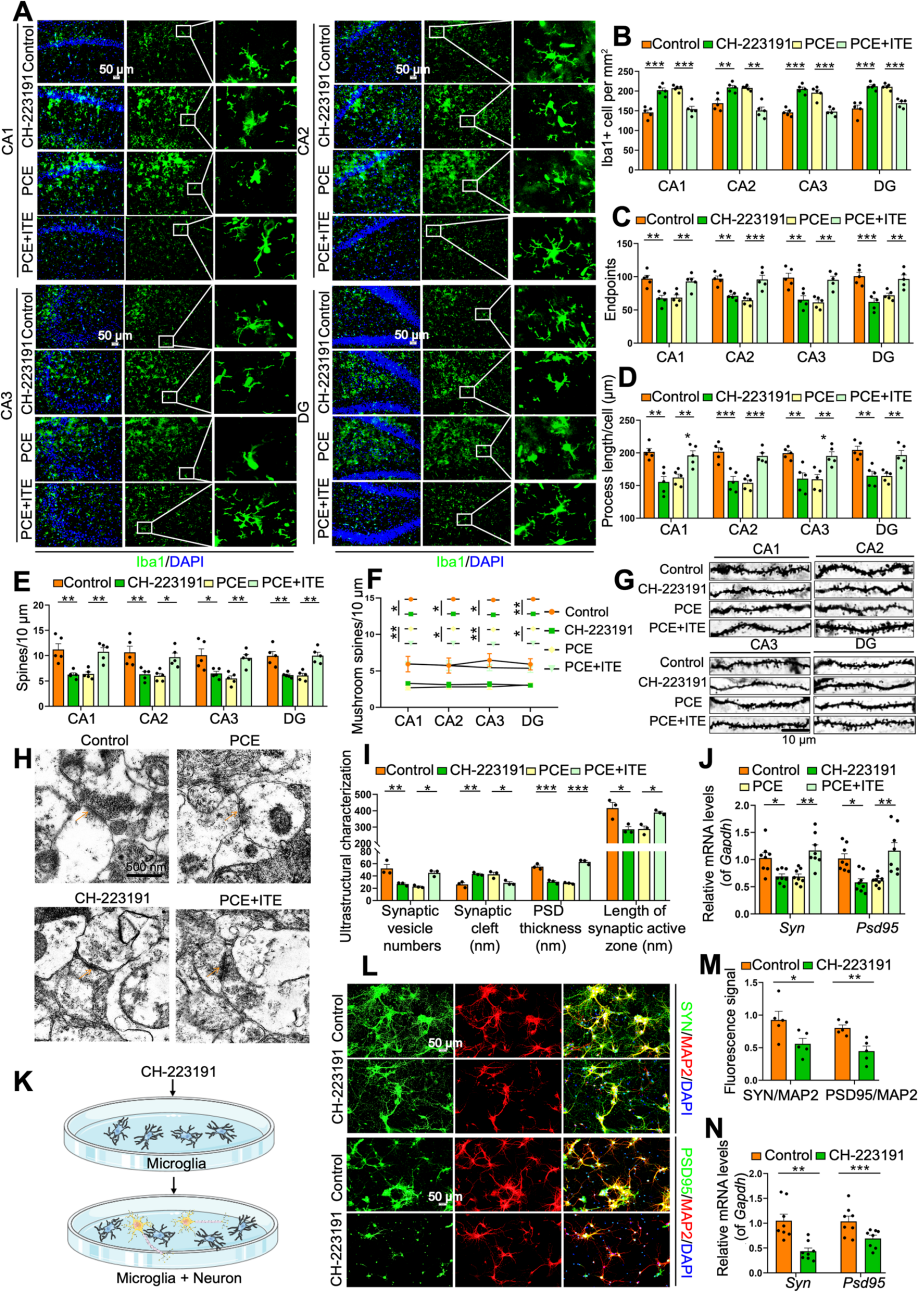
Dysregulated AHR/NF-κB signaling mediates hippocampal pathology both in the PCE-induced IUGR rats and in the co-cultured primary microglia and neurons. **A** Images under fluorescence microscopy showing different regions in hippocampus. Iba1 staining (green) and nuclear staining (DIPA, blue). Scale bar = 50 μm. **B**–**D** Number of Iba1^+^ cells, number of endpoints per microglia, and process length. **E** Total spine density in hippocampal neurons. **F** Mushroom spine density in hippocampal neurons. **G** Representative images of dendritic segments. Scale bar =10 μm. **H** Ultrastructure of synapses. Scale bar = 500 nm. **I** Synaptic vesicle numbers, synaptic cleft, postsynaptic density (PSD) thickness, and length of the synaptic active zone. **J** mRNA levels of *Syn* and *Psd95*. **K** Schematic of the experimental design in vitro. **L** Images under fluorescence microscopy showing co-cultured microglia and neurons. SYN staining (green), PSD95 staining (green), MAP2 staining (red), and nuclear staining (DIPA, blue). Scale bar = 50 μm. **M** Fluorescence signals of SYN^+^/MAP2^+^ and PSD95^+^/MAP2^+^. *N* mRNA levels of *Syn* and *Psd95*. Dots in panels represent individual samples. Data are presented as mean ± SEM. ^*^P < 0.05, ^**^P < 0.01, and ^***^P < 0.001

To confirm the role of AHR signaling in ASD susceptibility, we treated control rats with an AHR inhibitor (CH-223191) and IUGR rats with an AHR agonist (ITE) at PW4-6 (Fig. 2D). The CH-223191-treated control rats exhibited the aforementioned ASD-like symptoms, while the ASD-like symptoms of IUGR rats were recovered when treated with ITE (*P* < 0.05, *P* < 0.01, or *P* < 0.001, Fig. 2E-J). The CH-223191-treated control rats had inhibited AHR expression and promoted P-NF-κB in the hippocampus, whereas the ITE-treated IUGR rats had promoted AHR expression and inhibited P-NF-κB in the hippocampus (*P* < 0.05 or *P* < 0.01, Fig. 2K–M). Furthermore, control rats developed hippocampal microglia hyperactivation, synapse over-pruning, and synapse impairment after CH-223191 treatment. In contrast, ITE treatment reversed the above hippocampal pathology in IUGR rats (*P* < 0.05, *P* < 0.01, or *P* < 0.001, Fig. 3A–J, Fig. S3A-S3C).

We then extracted the primary rat hippocampal microglia, treated them with CH-223191, and mixed them with rat neurons to verify the role of AHR/NF-κB signaling in microglia function in vitro (Fig. 3K). When treated with the optimal dose of CH-223191, the hippocampal microglia showed a downregulated AHR expression as well as an upregulated P-NF-κB level (*P* < 0.05, *P* < 0.01, or *P* < 0.001, Fig. S3D-S3J). After the co-culture with CH-223191-treated microglia, the neurons exhibited a reduced complexity of dendrites (Fig. 3L), a decreased double-labeled immunofluorescence intensity of SYN+MAP2 or PSD95+MAP2 (*P* < 0.05 or *P* < 0.01, Fig. 3L and M), and a downregulated expression level of *Syn* and *Psd95* (*P* < 0.01 or *P* < 0.001, Fig. 3N), suggesting impaired synapses.

Based on the above in vitro and in vivo interventional experiments of AHR, we demonstrated that the AHR/NF-κB signaling mediated the hippocampal microglia hyperactivation, synapse over-pruning, and synapse impairment in the PCE-induced IUGR rats, which ultimately led to ASD susceptibility.

### Disturbed gut microbiota and reduced IPA production contribute to ASD susceptibility in PCE-induced IUGR rats

**Fig. 4:**
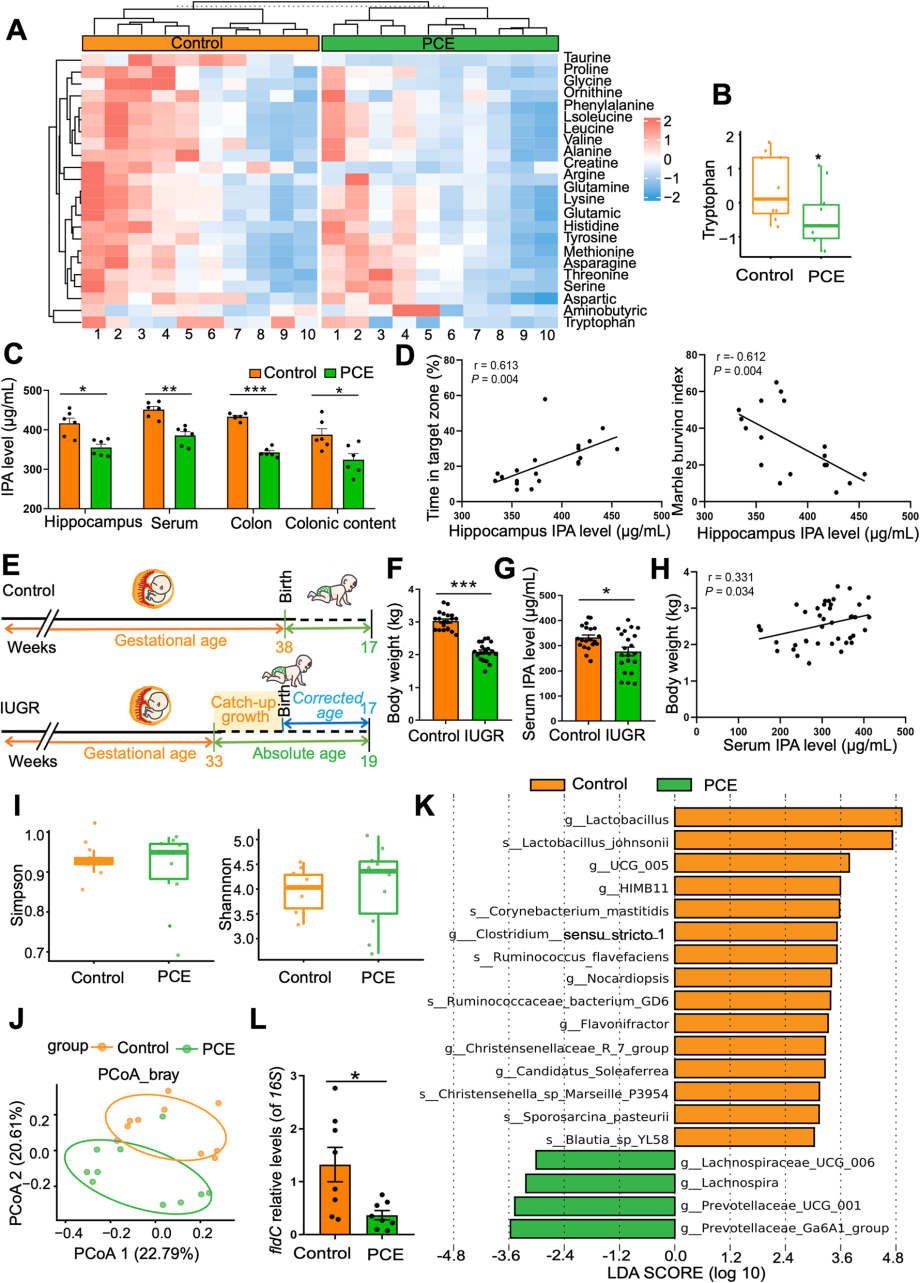
Disturbed gut microbiota and reduced IPA production in PCE-induced IUGR rats. **A** Heatmap of amino acids in colon content. **B** Tryptophan level in colon content. **C** IPA levels in different samples. **D** Correlations between the time spent in target zone or marble burying index and the IPA level in hippocampal tissue. **E** Schematic of the experimental design in infant cohort. **F** Body weight of the male infants. **G** Serum IPA level in the male infants. **H** Correlation between the serum IPA levels and the body weight of male infants. **I** Alpha diversity represented by the Simpson index and the Shannon index. **J** Beta diversity as shown by the PCoA plot. An ellipse represents the 68% confidence interval of microbial distribution in each group. **K** LEfSe of differentiating genera or species in gut microbiota between groups (LDA > 3). **L** DNA level of *fldC* in colon content. Dots in panels represent individual samples. Data are presented as mean ± SEM. ^*^P < 0.05, ^**^P < 0.01, and ^***^P < 0.001

Many tryptophan metabolites are endogenous ligands of AHR and can enter hippocampus by crossing the blood-brain barrier. Therefore, we explored whether alterations of tryptophan metabolites were involved in the ASD susceptibility of IUGR offspring. The metabolomic profiling of amino acids showed that tryptophan was significantly reduced in the intestinal content of the IUGR rats (*P* < 0.05, Fig. 4A, B), suggesting a concurrent change in tryptophan metabolism. We then focused on IPA, one of the main tryptophan metabolites, as our previous metabolomic study found a reduced serum level of IPA in these PCE-induced IUGR rats [37]. The enzyme-linked immunosorbent assays (ELISA) showed that the IPA levels in colon content (*P* < 0.05), colon tissue (*P* < 0.001), serum (*P* < 0.01), and hippocampal tissue (*P* < 0.05) were all significantly decreased in the IUGR rats (Fig. 4C). In the PCE-induced IUGR rats, we have found a positive correlation between the level of IPA and the activation of NF-κB in the hippocampus (*R* = 0.7614, *P* = 0.0006) (Fig. S4A). Moreover, the hippocampal IPA level was positively correlated with sociability (*R* = 0.613, *P* = 0.004) but negatively associated with stereotyped repetitive behaviors (*R* = −0.612, *P* = 0.004) (Fig. 4D). We also collected human samples and found that the serum IPA level of male IUGR infants was significantly lower at the mean adjusted age of 17 weeks compared to the male infants with a normal birth weight (*P* < 0.05 or *P* < 0.001, Fig. 4E–G). In addition, the serum IPA level was positively correlated with body weight in the IUGR infants (*R* = 0.331, *P* = 0.034, Fig. 4H). These results suggested that a decreased level of AHR ligand IPA is common in IUGR offspring.

IPA is a microbiota-specific tryptophan metabolite that cannot be produced by the host. To investigate whether the gut microbiota and its metabolite IPA are involved in the pathogenesis of IUGR-related ASD-like symptoms, we profiled the gut microbiota composition of IUGR rats and control rats at PW4 by 16s rDNA sequencing. Although the individual bacterial diversity (α diversity) of the IUGR rats was not different from the control rats (Fig. 4I), there was a significant difference in the population distribution (*β* diversity) of gut microbiota between the IUGR rats and the control rats (*R* = 0.33, *P* = 0.003, Fig. 4J). The linear discriminant analysis of effect size (LEfSe) analysis was used to reveal the major differences in bacterial genera and species between the IUGR rats and the control rats (LDA > 3 and *P* < 0.05, Fig. 4K). Among the altered bacterial taxa, *Clostridium*, which can convert tryptophan into IPA [46], was significantly reduced in the IUGR rats. The production of IPA depends on phenylacetate dehydratase, an enzyme expressed by the *fldC* gene [47]. We further found a significant reduction in the DNA level of *fldC* gene in the feces of the IUGR rats compared to the control rats (*P* < 0.05, Fig. 4L). In our study, we performed Spearman correlation analyses to investigate the relationship between the abundance of fecal *Clostridium* species and the frequency of ASD-like symptoms in PCE-induced IUGR rats. Our results indicate that a lower abundance of *Clostridium senso stricto 1* in fecal samples is correlated with reduced time spent by the rats in the targeted zone during the social choice test (*R* = 0.4909, *P* = 0.0279) (Fig. S4B). This suggests that the decreased levels of *Clostridium*, as well as its metabolite IPA, may contribute to the ASD-like symptoms observed in the PCE-induced IUGR rats.

**Fig. 5:**
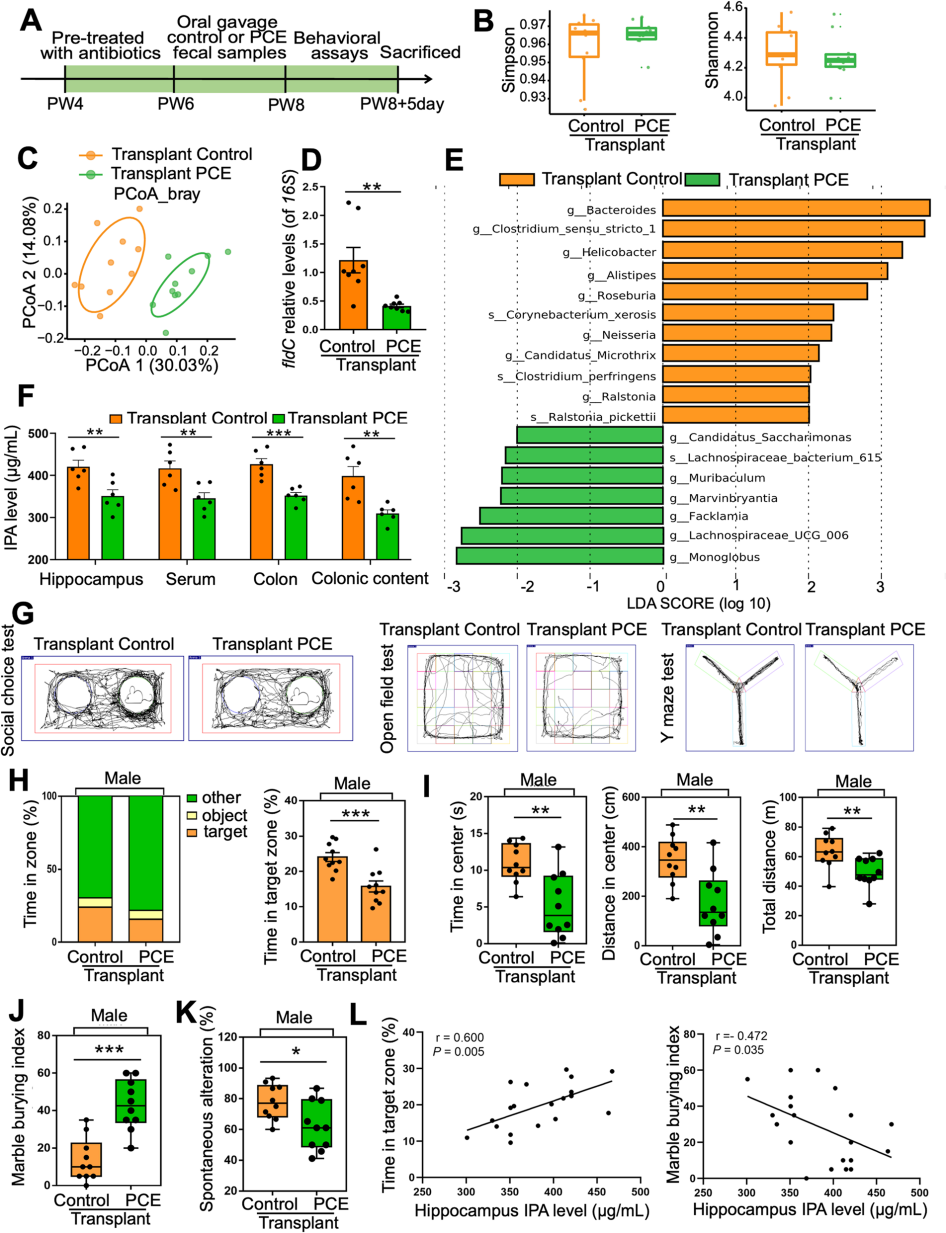
Disturbed gut microbiota mediates ASD susceptibility in PCE-induced IUGR rats. **A** Schematic of the fecal microbiota transplantation experiment. **B** Alpha diversity represented by the Simpson index and the Shannon index. **C** Beta diversity as shown by the PCoA plot. An ellipse represents the 68% confidence interval of microbial distribution in each group. **D** DNA level of *fldC* in feces*.* **E** LEfSe of differentiating genera or species in gut microbiota between groups (LDA > 2). **F** IPA levels in different samples. **G** An illustrative example of travel pathways of rats in social choice test, open field test, and Y maze test. **H** Time of rats spent in each zone in social choice test. **I** Time spent in the center, distance traveled in the center, and total distance traveled in open field test. **J** Marble burying index in marble burying test. **K** Spontaneous alteration rate in Y maze test. **L** Correlations between the time spent in target zone or marble burying index and the IPA level in hippocampal tissue. Dots in panels represent individual samples. Data are presented as mean ± SEM. ^*^P < 0.05, ^**^P < 0.01, and ^***^P < 0.001

Fecal microbiota transplantation (FMT) was then conducted to confirm the role of gut microbiota in IUGR-related ASD-like symptoms. The fecal microbiota suspension of IUGR rats and control rats at PW4 were intragastrically administered to antibiotic-treated pseudo-sterile rats at PW6-8 (Fig. 5A). Consistent with the donors (Fig. S4C-S4E), no significant difference was found in the individual bacterial diversity between groups in the recipient rats (Fig. 5B), whereas the microbial distributions were different between groups (*R* = 0.622, *P* = 0.001, Fig. 5C). The abundance of IPA-producing *Clostridium* and the level of *fldC* gene were both significantly decreased in the rats receiving IUGR fecal microbiota (*P* < 0.01, Fig. 5D, E). In addition, the IPA levels in colon content (*P* < 0.01), colon tissue (*P* < 0.001), serum (*P* < 0.01), and hippocampal tissue (*P* < 0.01) of the rats receiving IUGR fecal microbiota were significantly decreased (Fig. 5F). These results suggest that the fecal microbiota and its metabolic characteristics in IUGR rats have been successfully transplanted into the recipient rats.

After being transplanted with the IUGR fecal microbiota, the recipient rats developed ASD-like symptoms at PW8 (*P* < 0.05, *P* < 0.01, or* P* < 0.001, Fig. 5G–K). The hippocampal IPA level was again closely correlated with sociability (*R* = 0.600, *P* = 0.005) and stereotyped repetitive behaviors (*R* = −0.472, *P* = 0.035) (Fig. 5L). AHR expression was inhibited while P-NF-κB was upregulated in the hippocampus of the rats receiving IUGR fecal microbiota (*P* < 0.05 or *P* < 0.01, Fig. S5A, S5B). There was also hippocampal microglia hyperactivation (*P* < 0.05 or *P* < 0.01, Fig. S5C-S5F), synapse over-pruning (*P* < 0.05, *P* < 0.01 or *P* < 0.001, Fig. S6A-S6C), and synapse impairment (*P* < 0.05 or *P* < 0.01, Fig. S6D-S6I) in the rats receiving IUGR fecal microbiota.

Taken together, disturbed gut microbiota and reduced IPA production were found in the PCE-induced IUGR rats, and the FMT experiment demonstrated their contribution to the hippocampal pathology and the ASD-like symptoms in the PCE-induced IUGR rats by regulating the hippocampal AHR/NF-κB signaling.

### Postnatal IPA supplementation can reverse the ASD susceptibility in PCE-induced IUGR rats

**Fig. 6:**
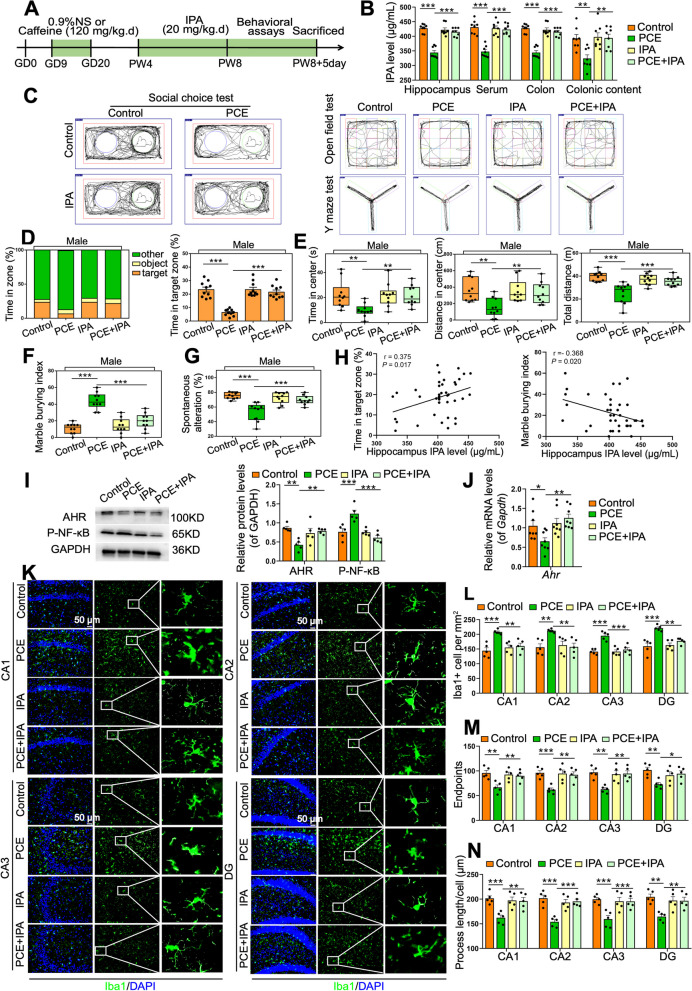
Postnatal IPA supplementation reverses the ASD susceptibility in PCE-induced IUGR rats. **A** Schematic of the oral IPA supplementation experiment. **B** IPA levels in different samples. **C** An illustrative example of travel pathways of rats in social choice test, open field test, and Y maze test. **D** Time of rats spent in each zone in social choice test. **E** Time spent in the center, distance traveled in the center, and total distance traveled in open field test. **F** Marble burying index in marble burying test. **G** Spontaneous alteration rate in Y maze test. **H** Correlations between the time spent in target zone or marble burying index and the IPA level in hippocampal tissue. **I** Protein levels of AHR and P-NF-κB. **J** mRNA level of *Ahr*. **K** Images under fluorescence microscopy showing different regions in hippocampus. Iba1 staining (green) and nuclear staining (DIPA, blue). Scale bar = 50 μm. **F**–**H** Number of Iba1^+^ cells, number of endpoints per microglia, and process length. **L** Total spine density in hippocampal neurons. **M** Mushroom spine density in hippocampal neurons. **N** Representative images of the dendritic segments. Scale bar =10 μm. Dots in panels represent individual samples. Data are presented as mean ± SEM. ^*^P < 0.05, ^**^P < 0.01, and ^***^P < 0.001

Since the reduction of IPA was closely associated with the ASD susceptibility in the PCE-induced IUGR rats, we then asked if IPA is protective for IUGR-related ASD-like symptoms. We gavaged the IUGR rats after weaning with a supplementary dosage of IPA (20 mg/kg) at PW4-8 (Fig. 6A). The IPA supplementation effectively recovered the deficiency of IPA in colon content, colon tissue, serum, and hippocampal tissue of the IUGR rats (*P* < 0.01, *P* < 0.001, Fig. 6B). The ASD-like symptoms of the IUGR rats were also significantly improved after IPA supplementation (*P* < 0.01, *P* < 0.001, Fig. 6C–G), and the hippocampal IPA level was closely correlated with sociability (*R* = 0.375, *P* = 0.017) and stereotyped repetitive behaviors (*R* = −0.368, *P* = 0.020) (Fig. 6H). The IPA supplementation reversed the AHR downregulation and the P-NF-κB upregulation in the hippocampus of IUGR rats (*P* < 0.05, *P* < 0.01, *P* < 0.001, Fig. 6I, J). Meanwhile, it also restored the hippocampal microglia hyperactivation, synapse over-pruning, and synapse impairment in the IUGR rats (*P* < 0.05, *P* < 0.01, or *P* < 0.001, Fig. 6K–N, Fig. S7A-S7I). These results suggest that postnatal IPA supplementation could effectively reverse the ASD susceptibility of PCE-induced IUGR rats, offering a novel avenue for the prevention and treatment of IUGR-related ASD-like symptoms.

## Discussion

The typical manifestations of IUGR are low birth weight, growth restriction, and multiple organ dysfunction [48, 49]. Epidemiological investigations suggested PCE as a clear trigger for IUGR [50, 51]. In our previous studies, we have established an IUGR rat model induced by PCE, which was successfully used in studying the developmental toxicology of multiple organs [22, 52–54]. In this study, both PCE-induced IUGR rats and IUGR infants were defined as having a birth weight less than 90% of the average birth weight of the control group. IUGR is a high-risk factor for ASD [55]. Multiple epidemiological surveys have demonstrated a significant increase in the incidence of ASD among children with low birth weight [56–58]. A retrospective survey of children aged 3–10 years old found that the risk of ASD in low birth weight individuals was approximately double that of normal weight individuals [56]. However, the underline mechanisms of the susceptibility to ASD in IUGR children has been under-studied. Based on the PCE-induced IUGR rat model, this study found that male IUGR offspring developed ASD-like symptoms in early childhood (PW4), as shown by stereotyped repetitive behaviors, social communication deficits, reduced free exploration, and reduced spatial memory.

Numerous studies have indicated that behavioral manifestations of ASD are associated with multiple brain regions, namely the amygdala, prefrontal cortex, and hippocampus [59–61]. Previous studies have shown that the amygdala and prefrontal cortex are mainly closely related to anxiety and social behavior of ASD, while the role of hippocampus in ASD behavior is relatively neglected. Bilateral hippocampal volumes are smaller in people with ASD than in healthy individuals, and hippocampal-dependent spatial reasoning and episodic memory are frequently impaired [62, 63]. Both clinical imaging evidence and animal studies supported that hippocampal lesions underlie the social memory deficits of ASD [39–41]. This study also revealed that learning memory and social memory, which are closely related to the hippocampus, were significantly impaired in IUGR offspring. The Y-maze experiment provides evidence supporting the crucial role of the hippocampus in spatial learning and memory abilities. Likewise, the social experiment emphasizes the significant association between social abilities and the hippocampal region, particularly the CA2 region, which is known to play a critical role in the processing of social cognitive memory [64]. Considering the hippocampus’s essential function in learning and memory, it is highly probable that the learning and memory processes observed in the Y-maze and social experiments in IUGR offspring are closely connected to molecular changes taking place in the hippocampus. Additionally, over-pruning of neuronal synapses by microglia was associated with social behavior defects [65], and reduced excitatory transmission in pyramidal neuronal synapses could cause ASD-like symptoms [66]. Therefore, we specifically examined the hippocampal changes of IUGR rats and focused on microglia and its influence on neuronal synapses. We found hippocampal microglia hyperactivation in the IUGR rats. Synapse over-pruning and synapse impairment were also found in the hippocampus of IUGR rats, as manifested by the reduction of mature synapses, the abnormal synaptic structure in the hippocampal CA2 region, and the downregulation of excitatory synaptic indicators. These findings indicated that the structural and functional changes of hippocampal microglia and neuronal synapses are the essential pathological basis for the ASD-like symptoms of male IUGR offspring.

ASD patients showed NF-κB activation in microglia, astrocytes, and neurons, especially in highly active microglia [67]. In addition, AHR loss could cause hippocampal-dependent memory impairments [68] and was associated with the severity of social communication disorders [69]. AHR can restrain NF-κB signaling through direct dimerization with the NF-κB subunits RelA and RelB [70]. It was reported that the inhibition of AHR expression resulted in NF-κB activation in macrophages [45]. Consistent with the above findings, our study revealed that the ASD-like symptoms in the male IUGR rats were accompanied by AHR downregulation and P-NF-κB upregulation in the hippocampus. Therefore, we speculate that abnormal AHR/NF-κB signaling in hippocampus might be the molecular basis of IUGR-related ASD-like symptoms. Through the AHR interventional experiments both in vitro and in vivo, we confirmed that the AHR inhibition led to the hippocampal pathological changes through the activation of NF-κB, ending up with the ASD susceptibility in the PCE-induced male IUGR rats.

New evidence provides support for the involvement of gut microbiota in regulating the core symptoms of ASD [71]. It has been suggested that gut microbiota variations are not associated with the diagnosis of ASD, and the reduced diversity of gut microbiota in patients with ASD could be a result of limited dietary variety [72]. However, these were the findings in children with ASD aged three and above. As a neurodevelopmental disorder, ASD undergoes a critical phase of disease formation during early neural development, specifically before the age of three [73]. Notably, the colonization of gut microbiota also occurs within this same timeframe [74]. Children with ASD show a reduced α-diversity of gut microbiota compared to healthy children before the age of three, while some recovery of gut microbiota changes can be observed in ASD children after this age [75]. Furthermore, transplanting gut microbiota from ASD patients into germ-free mice shortly after weaning showed that the colonization of ASD gut microbiota is sufficient to induce characteristic ASD-like symptoms [76]. Hence, it is highly probable that abnormal gut microbiota colonization in early life stages plays an essential role in the onset and progression of ASD. The establishment of gut microbiota could be affected by various intrauterine factors, such as a high-fat diet, antibiotic use, infection, and stress during pregnancy [77–79]. In this study, we found that the ASD-like symptoms of PCE-induced male IUGR rats were accompanied by alterations in gut microbiota composition and reduction of its metabolite IPA. Furthermore, a study has reported a decrease in the abundance of *Clostridium* species in fecal samples of individuals with ASD compared to neurotypical controls [80]. In line with this, we observed a significant decrease in the abundance of *Clostridium*, which converts tryptophan into IPA, in the PCE-induced IUGR rats. This change is closely associated with the ASD-like symptoms observed. FMT from the IUGR rats into the pseudo-sterile rats could not only recapitulate the ASD-like symptoms but also modulate the AHR/NF-κB signaling in hippocampus. Therefore, the disturbed gut microbiota and its metabolism were considered an essential contributing factor to the ASD with a fetal origin.

IPA is an indole metabolite specifically produced by the tryptophan metabolism of gut microbiota. It can cross the blood-brain barrier and act as an endogenous AHR ligand to activate AHR signaling, thus playing a regulatory role in the central nervous system. We found decreased serum IPA levels both in the PCE-induced IUGR rats and the IUGR infants, suggesting that a reduced IPA level is a common phenomenon of IUGR. Of note, the IPA levels in colon content, colon tissue, serum, and hippocampal tissue of IUGR rats were all decreased, and the hippocampal IPA level was negatively correlated with the core ASD-like symptoms, indicating a protective role of IPA in the IUGR-related ASD-like symptoms. Indeed, IPA has already been suggested to have a neuroprotective effect [81]. To confirm the postulation, we gave the PCE-induced male IUGR rats a 4-week oral supplementation of IPA after weaning. We found that IPA, as an AHR ligand, reversed the hippocampal microglial activation and synaptic over-pruning in the IUGR rats by modulating the AHR/NF-κB signaling, thereby restoring their ASD-like symptoms. Furthermore, IPA has a higher affinity for human AHR than rodent AHR [82]. Therefore, supplementation of gut microbiota-derived IPA could be a novel choice for the prevention and treatment of ASD with a fetal origin. After administering 20 mg/kg.d IPA to normal rats, we observed an average serum concentration of 2.26 mM (equivalent to 358.78 μM in humans), which was significantly higher than the previously reported average serum concentration of IPA in healthy adults (~1.0 μM) [82]. The dosage of 20 mg/kg.d IPA in rats corresponds to a daily intake of 190 mg IPA for a 60 kg human, which can be achieved through dietary supplementation. Although direct supplementation of 20 mg/kg.d IPA has been proven safe and neuroprotective in rats, further investigation is needed to determine the appropriate dosage for human use.

Our study had certain limitations. Firstly, this study only focused on the ASD susceptibility and related mechanisms in male IUGR offspring, owing to the sex bias of ASD. Future research taking into account gender differences will deepen the understanding of ASD with a fetal origin. Secondly, given that the mechanisms of IUGR are complex and animal models for IUGR are diverse, experiments on other IUGR models and data from the fecal samples of IUGR patients will be needed to further verify the therapeutic role of IPA on IUGR-related ASD. This study primarily focused on the hippocampal changes associated with social memory deficits in ASD, while lacking information on brain regions linked to anxiety (e.g., the amygdala) and social behavior (e.g., the prefrontal cortex). Future mechanistic research should aim to investigate whether the observed molecular changes are specific to the hippocampus in ASD or if they extend to other brain regions. Finally, we lack the follow-up data to know whether the reduced serum IPA level in the IUGR infants will be associated with ASD-typical behavioural deficits when they get older. Future research taking into account follow-up data will deepen the explanation of the relationship between IPA and ASD-typical behavioural deficits.

## Conclusion

**Fig. 7:**
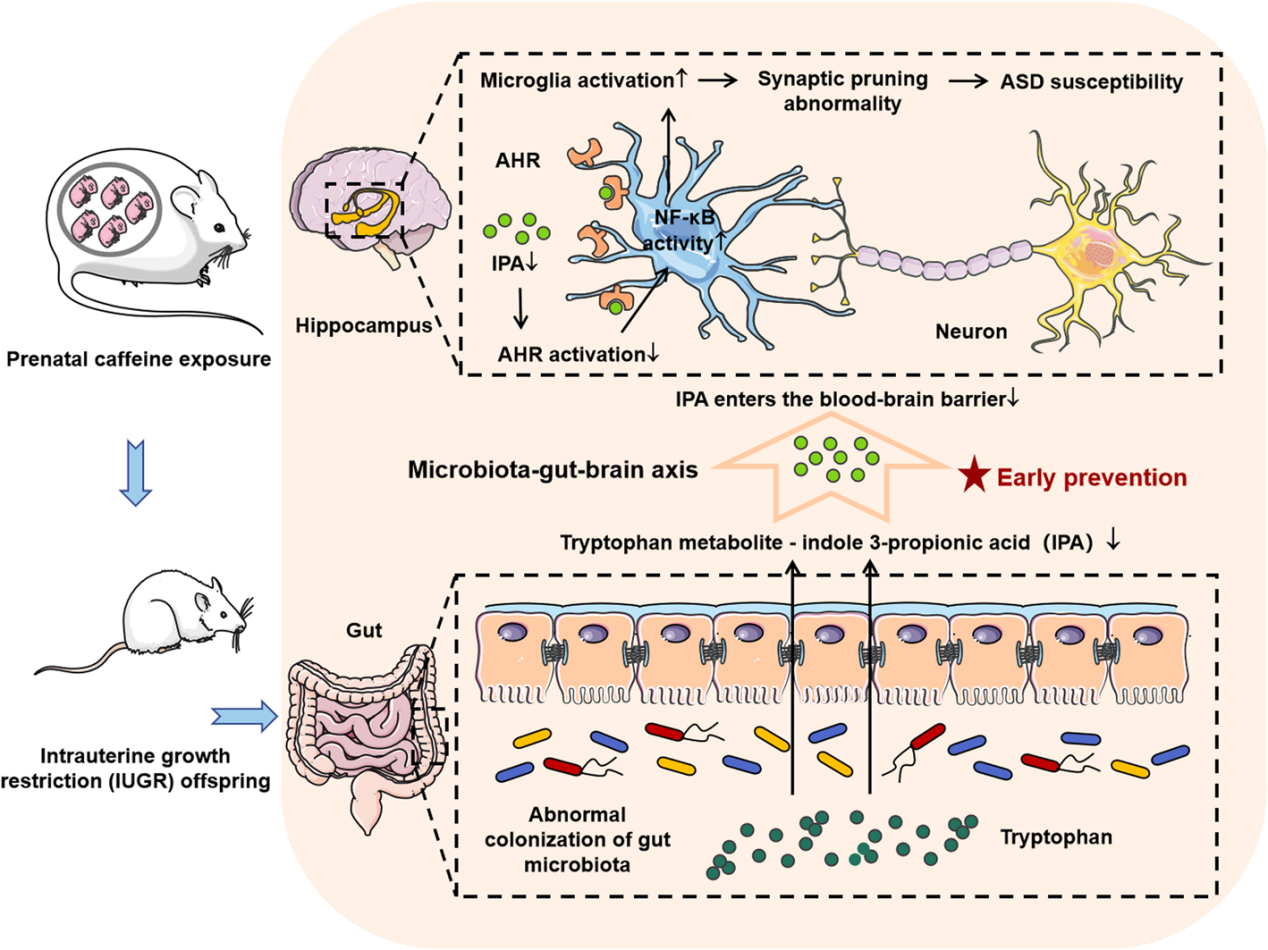
The microbiota-IPA-brain axis regulats the ASD susceptibility in PCE-induced IUGR offspring, and supplementation of microbiota-derived IPA might be a promising interventional strategy for ASD with a fetal origin

We verified in this study that the disturbed gut microbiota and the reduced production of its metabolites IPA in the PCE-induced male IUGR rats led to a reduced amount of IPA entering hippocampus through peripheral circulation, resulting in a dysregulated hippocampal IPA/AHR/NF-κB signaling. This is followed by hippocampal microglia hyperactivation and neuronal synapse over-pruning, which finally ends up with ASD-like manifestations. Furthermore, oral IPA supplementation in the postnatal period can effectively reverse the IUGR-related ASD pathology and manifestations (Fig. 7). Our study uncovered the role of the gut microbiota-IPA-brain axis in IUGR-related ASD-like symptoms and proposed that the supplementation of IPA, a gut microbiota-specific derivative, might be a promising strategy for the prevention and treatment of ASD with a fetal origin. Our study also accentuated the importance of avoiding the stimulation of adverse environmental factors during pregnancy and ensuring the development of the gut-brain axis during childhood.

## Materials and methods

### IUGR rat model

The animal study has been approved by the Animal Experiment Ethics Committee of Wuhan University (Permit No. WP20210476). All operating procedures were carried out following the relevant regulations of the China Animal Welfare Protection Association for the use of experimental animals.

SPF grade Wistar rats (female 240 g/male 350 g, 10–11 weeks old) were purchased from Beijing Speifu Biotechnology Co., Ltd. All rats were adaptively fed for 1 week under standard conditions (room temperature: 18–22 °C; humidity: 40–60%; light cycle: light-dark cycle for 12 h). Then, females and males were caged at a ratio of 2:1 at 18:00 every night. Vaginal secretions were taken for microscopic examination the next morning, the appearance of sperm in vaginal smears confirmed mating, and the day of mating was designated as the gestational day (GD) 0. Pregnant rats were divided into the control and PCE groups. To establish a stable animal model of IUGR and avoid early abortion and stillbirth, pregnant mice were gavage with 120 mg/kg.d caffeine (Sigma, USA) during GD9-20 according to our previous study [21, 22, 83]. The control group was intragastrically administered an equal volume of normal saline. Pregnant rats gave birth naturally, and a pup size of 10 to 14 was considered eligible (*n*=10 pregnant rats per group). The male and female offspring were distinguighed, weighed, and evaluated if they had IUGR (defined as weighted less than 10% of the average weight of neonates in the control group) on the first day of birth [52]. The offspring rats were labeled and routinely fed. After weaning (PW4), 5 male normal-weight offspring rats from the control group and 3 male IUGR offspring rats from the PCE group in each litter were kept. One male offspring rat from each litter of two groups was randomly selected at PW4 to undertake the behavior tests (*n*=10 per group). The rats were then anesthetized with 2% isoflurane and euthanized. The serum, hippocampus, colon, and colon contents were collected for morphological and molecular biology tests. The remaining qualified offspring were used for further interventional experiments.

### Human cohort

Approved by the Medical Ethics Committee of Wuhan University (Permit No. WHU2021-jc015), 21 male IUGR infants and 20 healthy male infants aged 0 to 1 year old were recruited at the Affiliated Hospital of Wuhan University. Written informed consent was obtained from a family member. The serum of infants was collected and stored at –80 °C immediately. The inclusion criteria were ① singleton pregnancy, ② gestational age within 28–40 weeks, and ③ the birth weight of the control infants was suitable for their gestational age, while the birth weight of the IUGR infants was lower than the tenth percentile of infant birth weight in the same period [52]. Infants with structural, genetic, or chromosomal abnormalities or congenital genetic disorders were excluded. In this study, the mean gestational age of normal birth weight infants and IUGR infants were 38 weeks and 33 weeks, respectively. The birth weight of infants with corrected age is more suitable for evaluating developmental problems, especially motor development and intellectual development [84, 85]. Compared with their full-term counterparts, preterm infants could easily be misestimated at risk of developmental impairment if the gestational age was not corrected and the postnatal catch-up growth process was ignored [86]. At the time of serum collection, the mean corrected age of the infants in the two groups was both 17 weeks after birth (Supplementary Table S1).

### Rat interventional experiments

To avoid being confounded by maternal lactation, PW4 was selected as the starting timepoint for all the interventions. For the study of activation or inhibition of AHR signal, one normal-weight and one IUGR male rat at PW4 from different litters (*n*=10 per group) were randomly selected to be given 10 mg/kg.d AHR inhibitor CH-223191 (MedChemExpress, USA) [87] or 10 mg/kg.d AHR agonist ITE (MedChemExpress, USA) [88] by intraperitoneal injection for 2 weeks. For the IPA supplementation experiment, one normal-weight and one IUGR male rat at PW4 from different litters (*n*=10 per group) were randomly selected to be given 20 mg/kg.d IPA (Aladdin, China) by gavage for 4 weeks [89]. Previous studies have shown that oral administration of 20 mg/kg.d of IPA in rats does not result in significant toxic or adverse effects. In fact, it has even demonstrated certain neuroprotective properties [46, 90]. As a result, we selected a dosage of 20 mg/kg.d of IPA for our interventional experiments in PCE rats. Tryptophan is an important source of IPA. All rats were fed a maintenance diet consisting of 0.2% of tryptophan (WQJX BIO-TECHNOLOGY, China). The average daily food intake of the offspring rats was recorded, and no significant differences were found among the different groups (Fig. S8A). Therefore, the background tryptophan intake was comparable between the PCE rats gavaged with IPA and the PCE rats gavaged with solvent. The background tryptophan intake probably did not affect exploring the role of IPA gavage in improving ASD-like symptoms in PCE-induced IUGR rats.

For the fecal microbiota transplantation (FMT) experiment, we selected 10 PCE-induced IUGR male rats and 10 normal-weight male rats as donors. In each group, 0.3 g of feces from each rat were pooled together to obtain a total quantity of 3 g. The fecal mixture was then dissolved in 10 ml of sterile PBS. Next, the solution was filtered through a 100-μm-cell filter to obtain a suspension of bacterial cells. To maintain the viability of the anaerobic bacteria, the fecal preparation was carried out inside an anaerobic chamber. Regarding the recipients, 20 normal-weight male rats at PW4 were gavaged with the antibiotic mixture (1 mg/ml neomycin, 1 mg/ml ampicillin, 0.5 mg/ml metronidazole, and 0.5 mg/ml vancomycin) for 2 weeks to establish the pseudo-sterile rat model [91]. In our study, we did compare the bacterial colony counts in the feces of SPF rats treated with antibiotics and those not treated with antibiotics. Specifically, we collected rat feces from the same group, mixed them in equal amounts, and dissolved the mixed sample in normal saline at a ratio of 1 g : 100 ml. Then, we spread 20 μl of the diluted sample evenly on the brain heart infusion broth medium agar plates. The plates were then incubated in an anaerobic incubator at 37 ℃, with the plates placed upside down for 24 h once they dried. Finally, the colony-forming units (CFUs) were calculated and compared between the two groups. In result, the number of CFUs of the pseudo-sterile rat was significantly lower than that of SPF rats, indicating the successful establishment of pseudo-sterile rat (Fig. S8B). They were then separated into two groups (*n* = 10 per group). The recipient pseudo-sterile rats were gavaged with 200 μl of the above fecal suspension from normal-weight or IUGR male rats every day for 2 weeks. In our study, prior to transplantation, the recipient rats were given ample opportunities for social interaction before PW6. To ensure consistency in the isolation process among groups, the recipient rats in the control group were also housed separately during transplantation. Therefore, the isolation procedure should not have affected the impact of fecal transplantation on the ASD-like symptoms observed in the recipient rats in our experiments.

After the interventions, the rats were tested for ASD-like behaviors and then anesthetized with 2% isoflurane and euthanized. The serum, hippocampus, colon, and colon contents were collected for morphological and molecular biology tests.

### Co-culture of microglia and neurons

The whole hippocampal tissue was isolated from the newborn Wistar rats (1–3 days), cut into pieces, and digested with 0.125% EDTA-trypsin (Gibco, USA) at 37 °C for 20 min. After the digestion was terminated, the suspension was collected and centrifuged, and then, the supernatant was discarded. The cells were resuspended in DMEM high glucose medium containing 10% FBS and 1% penicillin-streptomycin and then filtered and seeded into 75 cm^2^ cell culture flasks. The cells were cultured in an incubator at 37 °C under 5% CO_2_, and the medium was changed once and every 3 days thereafter. After 1 week, the cell culture flasks were fixed on a constant temperature shaker at 37 ℃, 80–200 r/min and shaken for 2 h. Purified microglia were obtained by resuspension and verified by immunofluorescence staining.

The whole hippocampal tissue was isolated from the newborn Wistar rats (1–3 days), cut into pieces, and digested using papain for 30 min, followed by digestion termination with fetal bovine serum, filtration, centrifugation, and resuspension, successively. The cells of neurons were seeded on glass slides or six-well plates coated with poly-L-lysine at a concentration of 10^6^/ml and cultured in an incubator at 37 °C under 5% CO_2_. After 6 h, the medium was replaced with the serum-free neurobasal one (containing 2% B27). The growth of neurons was observed under a microscope and recorded.

As to the co-culture, the purified primary microglia were pretreated with 10 μM CH-223191 (MedChemExpress, USA) for 2 h and then inoculated at a density of 1×10^5^/ml in a six-well plate containing primary neurons. Neurons and microglia were distinguished by immunofluorescence staining. Cytotoxicity was quantified using the CCK-8 kit for the selection of optimal concentration and time for CH-223191 treatment. Briefly, 100 μl of the microglia suspension was seeded onto a 96-well plate at a concentration of 5×10^3^/well. The cells were incubated at 37°C and treated with 0, 5, 10, 20, 40, or 80 μM CH-223191 for 2 h or with 10 μM CH-223191 for 0, 0.5, 1, 2, 4, or 8 h. The absorbance at 450 nm was measured by a microplate reader to calculate the cell viability.

### Behavioral assays

The rats were kept in the experimental room for at least 30 min for acclimatization prior to the behavioral tests. The less stressful behavioral tests were performed first, with more stressful ones done subsequently. The order of the tests was as follows.

#### Open field test

The field experiment was illuminated using artificial lighting, a 40W incandescent bulb positioned 2.8 m above the center of the field. The rats were placed within a testing arena for 3 min for acclimatization and then tested for 5 min. The experimental instrument was cleaned with 75% ethanol between two rats. The SMART video tracking system (SMART v3.0, Panlab, Spain) was employed to record and analyze the exploratory tracks, the total distance moved, and the distance moved and the time taken within the central zone of the rats.

#### Y maze test

The rats were put in a spontaneous alternating Y-maze to assess short-term spatial memory. The Y-maze device was made of polypropylene, and the length, width, and height were 50 cm, 10 cm, and 20 cm, respectively. The 3 different branches in a Y-maze are known as 3 arms (A, B, and C). The rats were placed at the end of the same arm and left to move freely for 8 min. The SMART video tracking system was employed to record and analyze the rats’ exploratory tracks. In general, a rat should prefer to study a new arm in the maze rather than return to an arm that it visited before. The three consecutive choices into different arms is considered a correct alternation, such as ABC, BCA, and CAB. Finally, the free alternation rate was calculated as [correct alternation times/(total times of arm entry-2)]*100%.

#### Social choice test

The test was performed in the open-field arena. A young stranger rat in a wire mesh cage was used as a social cue. A caged object and a caged stranger rat were placed simultaneously on the opposite side of the arena. The test rat was then allowed to explore either the caged object or the caged stranger rat, and its movement was videotaped. The recorded video file was analyzed by the SMART video tracking system. The time spent in the corner proximal to the caged stranger rat was measured.

#### Marble burying test

The percentage of marbles buried was used as an index for stereotyped behavior. The rats were placed in a testing arena (arena size: 80 × 40 cm^2^, bedding depth: 5 cm) containing 20 glass marbles (equidistant from each other in an arrangement style of 4 × 5). At the end of the 15 min exploration period, the rats were removed from the testing cages, and the number of buried marbles was recorded. The criterion for a buried marble was more than 50% of the marble surface covered by the bedding material.

### Transcriptome analysis

The transcriptomic data of hippocampal tissue in a classical ASD mouse model induced by prenatal valproic acid exposure was retrieved from the GEO database (GSE180564). The differential genes were analyzed by edgeR. The signal channel enrichment and the gene set enrichment analysis (GSEA) were performed using the R package ClusterProfiler in R (Version 3.6.3).

### Gut microbiota profiling

The V3-V4 region of the bacteria’s 16S rRNA gene was amplified by PCR with barcode-indexed primers (338F and 806R) using FastPfu Polymerase. Amplicons were then purified by gel extraction and quantified using QuantiFluor-ST. The purified amplicons were pooled in equimolar concentrations, and paired-end sequencing was performed using an Illumina MiSeq instrument (Illumina, USA). The raw sequencing data were separated into individual samples based on barcodes. Subsequently, the barcodes and primers were removed, and the data was assembled using the FLASH software (version 250.1.2). The assembled tags underwent quality control to obtain clean tags, which were further filtered to eliminate chimeric sequences, resulting in a set of effective tags suitable for subsequent analysis. Regarding taxonomic annotation, the effective tags from all samples were clustered into operational taxonomic units (OTUs) using a 97% identity threshold by QIIME (version 1.8.0). The sequences of the OTUs were then annotated to identify different taxa by Mothur (version v.1.30). The species annotation results were visualized using KRONA for visualization. The community structure of gut microbiota was assessed by *α* and *β* diversity. The Shannon index and Simpson index were used to evaluate *α*-diversity. Wilcoxon’s rank-sum tests were used to determine the significance of differences in *α* diversity between groups. The *β* diversity was calculated based on the Bray-Curtis distances and displayed as principal coordinate analysis (PCoA) plots. Permutational multivariate analysis of variance (PERMANOVA) was conducted to determine the significance of differences in *β* diversity between groups. The *R* value refers to the extent to which a variable can explain the *β* diversity of the microbial communities. LEfSe was carried out to identify discriminating bacterial taxa between groups. The tests listed above were conducted on *R* (Version 3.6.3) or on the cloud platform of Novogene Biology Information Technology Co., Ltd. (https://magic.novogene.com/).

### Fecal amino acid profiling

Amino acids in frozen fecal samples were determined by liquid chromatography-tandem mass spectrometry (LC-MS/MS). In brief, 50 mg of lyophilized feces was homogenized with 600 μl of ultrapure water. The extracts were centrifuged at 13,000 rpm and 4 °C for 10 min, and the supernatant retrieved was immediately transferred. The residue of the previous extraction was further extracted with 600 μl of ice-cold methanol to obtain another supernatant. The first and second supernatant were mixed in equal volumes, and 80 μl of the mixture was precipitated with 80 μl of methanol, which contains chloro-d-phenylalanine (100 ng/ml) and ketoprofen (10 ng/ml). After homogenization and centrifugation, 100 μl of the final supernatant was transferred to a sample vial for LC-MS/MS analysis.

### Transmission electron microscopy analyses

The hippocampal tissue was cut into 1 mm^3^ and successively fixed in 2.5% glutaraldehyde and 1% osmium acid. The samples were then rinsed using 0.1 M phosphoric acid and dehydrated step by step using ethanol before being embedded and sliced. The ultrastructural changes of neurons were observed under the transmission electron microscope (Hitachi, Japan) after the sections were counterstained by 3% uranium acetate-citric acid. Image analysis was performed using Image-Pro Plus software (version 6.0, Media Cybernetics Inc, MD).

### Golgi staining and analysis

Golgi-Cox staining was performed using the FD Rapid GolgiStain^TM^ Kit (FD NeuroTechnologies, USA) [92]. Briefly, the rats’ brains were immersed in the Golgi-Cox solution for 14 days, transferred to the 30% sucrose solution, and incubated for 2–5 days in the dark at room temperature. The serial coronal sections (100 µm thick) were obtained by a Leica VT1000S vibratome, mounted on the Superfrost plus slides (Thermo Fisher Scientific, USA), stained by working liquid, and coverslipped. The branched total dendritic length was determined using Neuroexplorer software and Sholl analysis. Sholl analysis was performed by counting the dendrites traversing a series of concentric circles at 10-µm intervals from the cell body. To calculate the dendritic spine density in the hippocampal CA1, CA2, CA3, and DG regions, the number of spinous processes in a random 10 µm-long dendrite segment was counted. The dendritic spine density was determined by dividing the number of spinous processes in the random dendrite segment by its length. The dendritic spines are classified into mushroom (protrusions with a small neck and a large head), stubby (protrusions tightly connected to the dendritic shaft and lack of a well-defined channel), and slight (longbows with a bulbous head). The mushroom spine density was determined by dividing the number of spinous processes in a random dendrite segment of a mushroom dendritic spine by its length.

### Immunofluorescence staining analysis

The brain tissue of rats was collected, fixed with 4% paraformaldehyde, immersed in sucrose solutions (20%, 25%, and 30%, sequentially), and preserved at 4 °C. The brain tissue was then embedded in the Tissue Tek O.C.T. compound (Sakura Finetek, USA), and continuous coronal slices (10 μm) were prepared for immunofluorescence. After antigen retrieval and blocking using 10% sheep serum, the slices were incubated with the primary antibodies of rabbit-derived Iba1 monoclonal antibody (1:500, Abcam, USA), FITC anti-rabbit IgG (1:200, ABclonal, China), and DIPA mixture, sequentially. The images were taken using a fluorescence microscope (Olympus, Japan). The activation of microglia was determined using the Image-Pro Plus software (version 6.0, Media Cybernetics Inc., MD) and Sholl analysis. To assess the activation of microglia in the hippocampal CA1, CA2, CA3, and DG regions, the number of microglia, the number of endpoints per microglia, and the length of processes were quantified. Five offspring rats from five different litters were randomly assigned to each group.

The primary microglia and neurons were washed with cold PBS and fixed with paraformaldehyde. Then, the cells were blocked in 10% sheep serum and incubated with the following primary antibodies overnight at 4 °C: rabbit-derived Iba1 polyclonal antibody (1:500, Abcam, USA), mouse MAP2 monoclonal antibody (1:150, Abcam, USA), rabbit PSD95 monoclonal antibody (1:200, Abcam, USA), and rabbit SYN polyclonal antibody (1:200, Abcam, USA). After rinsing, the cells were incubated with Cy3-labeled goat anti-rabbit IgG (1:200, Thermo Fisher Scientific, USA) and Alexa Fluor 488-labeled goat anti-mouse IgG (1:200, Thermo Fisher Scientific, USA), followed by subsequent DIPA staining. The images were taken using a fluorescence microscope and analyzed by the Image-Pro Plus software.

### Western blotting

The total proteins were extracted from the rats’ hippocampus using the RIPA lysate containing PMSF and separated by 10% SDS-PAGE. The proteins were transferred onto PVDF (Millipore, USA) membranes, blocked with 5% skim milk, and then incubated at 4 °C overnight between the membranes with the following primary antibodies: rabbit AHR polyclonal antibody (1:100, ABclonal, China), rabbit NF-κB polyclonal antibody (1:100, Cell Signaling Technology, USA), and mouse GAPDH (1:2000, ABclonal, China). After rinsing, the membranes were then incubated with goat-anti-mouse IgG or goat-anti-rabbit IgG at room temperature for 2 h. The ECL chemiluminescence was performed using a chemiluminescence gel imaging system (Tanon, China), and the protein band density was analyzed by the Image-Pro Plus software (version 6.0, Media Cybernetics Inc., MD). Five offspring rats from five different litters were randomly assigned to each group.

### IPA quantification

Colon content, colon tissue, and hippocampal tissue of the rats were collected and homogenated, and the homogenate was centrifuged at 3000 rpm for 3 min to obtain the supernatant. The serum of rats and human infants was also collected. An ELISA kit (Meimian, China) was used to detect the IPA levels in the above samples, according to the manufacturer’s protocol. The concentrations were calculated on the basis of OD values at 450 nm and the standard curves.

### Real-time quantitative PCR (RT-qPCR)

The total RNA was extracted from the rats’ hippocampal tissue using TRIzol reagent (Invitrogen, USA) and converted into cDNA using HiScript II Select qRT SuperMix (Vazyme, China) according to the supplier’s instructions. The cDNA products were kept at −20 °C prior to use. The mRNA levels were quantified in triplicate by RT-qPCR with SYBR premix (Vazyme, China) on a CFX Manager 3.1 system (Bio-Rad, USA). The mRNA levels were evaluated by the 2^-△△Ct^ method. *Gapdh* was chosen as the internal reference genes [93]. The primers used for RT-qPCR were presented in Supplementary Table S2.

### fldC gene detection

The genomic DNA was extracted from the rats’ colonic content using the TIANamp Stool DNA Kit (TIANGEN, China) and subjected to RT-qPCR for the detection of *fldC*. The primers used for *fldC* detection were 311F-TGGGGAATATGATATGTTGTCTGGCATGATG and 311R-TGTTCAGCTAATCTATCCATTGGTGTATTCGC [47]. The 16S rDNA was used as the internal reference.

### Statistical analysis

The statistical analyses were performed using SPSS 22.0 (Science Inc., USA), GraphPad Prism 8.0 (GraphPad Software, USA), or R 3.6.3 software (http://www.r-project.org). The experimental data was presented as mean ± SEM. All data were checked for normality using Shapiro-Wilk’s test and for homogeneity of variance using Levene’s test.

Two-tailed unpaired *t* tests were used to compare the data between two independent groups. One-way ANOVA with Tukey’s multiple comparison post hoc test was usd to compare the data within more than two separate groups. Two-way ANOVA with Sidak’s multiple comparison post hoc test was used to analyze the two-factor data. The correlations were evaluated by the Spearman correlation coefficient. *P* values less than 0.05 were considered statistically significant.

### Supplementary Information

**Additional file 1: Fig. S1.** PCE-induced IUGR female rats did not show typical ASD-like manifestations. (A, C) Fetal body weight on the first day after birth. (B, D) IUGR rate. (E) An illustrative example of travel pathways of female rats in social choice test, open field test, and Y maze test. (F) Time of female rats spent in each zone in social choice test. (G) Marble burying index in marble burying test. (H) Time spent in the center, distance traveled in the center, and total distance traveled in open field test. (I) Spontaneous alteration rate in Y maze test. Dots in panels represent individual samples. Data are presented as mean ± SEM. ^*^P < 0.05, ^***^P< 0.01. **Fig. S2.** AHR/NF-κB signaling is involved in ASD. (A) The top enriched pathways of differentially expressed genes by Clusterprofiler. (B) Gene set enrichment analysis (GSEA) plots shows NF-κB signaling pathway. **Fig. S3.** AHR intervention induces abnormalities of neurons and activation of microglia in the co-cultured primary hippocampal microglia and neurons in vitro. (A) Representative reconstruction of hippocampal neurons. Scale bar = 50 μm. (B) Dendritic length of hippocampal neurons. (C) Number of branch points in hippocampal neurons. (D) Dose-effect: cell viability after treated with different concentrations of CH-223191 (0, 5, 10, 20, 40 or 80 μM) for 2 h. (E) Time-effect: cell viability after treated with 10 μM CH-223191 at different time (0, 0.5, 1, 2, 4, and 8 h). (F) Protein levels of AHR and P-NF-κB. (G) mRNA levels of *Ahr*. (H) Morphology of primary microglia under the light microscope. Scale bar = 50 μm. (I) Morphology of primary microglia under the fluorescence microscope. Iba1 staining (green) and nuclear staining (DIPA, blue). The arrows point at activated microglia. Scale bar = 50 μm. (J) Microglia activation rate. Dots in panels represent individual samples. Data are presented as mean ± SEM. ^*^P < 0.05, ^**^P< 0.01, ^***^P < 0.001. **Fig. S4.** Correlation analysis and gut microbiota signatures of donor rats. (A) Spearman correlation between the IPA level and the P-NF-κB level in hippocampal tissue. (B) Correlations between the time spent in target zone and the *Clostridium* abundance. The correlations were evaluated by the Spearman correlation coefficient. (C) Alpha diversity represented by the Simpson index and the Shannon index. (D) Beta diversity as shown by the PCoA plot. An ellipse represents the 68% confidence interval of microbial distribution in each group. (E) LEfSe of differentiating genera or species in gut microbiota between groups (LDA > 2). Dots in panels represent individual samples. **Fig. S5.** Transplantation of microbiota from the IUGR rats causes dysregulation of AHR/NF-κB signaling and activation of hippocampal microglia. (A) Protein levels of AHR and P-NF-κB. (B) mRNA levels of *Ahr*. (C) Images under fluorescence microscopy showing different regions in hippocampus. Iba1 staining (green) and nuclear staining (DIPA, blue). Scale bar = 50 μm. (D-F) Number of Iba1^+^cells, number of endpoints per microglia., and process length. Dots in panels represent individual samples. Data are presented as mean ± SEM. ^*^P < 0.05, ^**^P < 0.01, ^***^P < 0.001. **Fig. S6.** Transplantation of microbiota from the IUGR rats causes abnormalities of hippocampal neurons. (A) Representative reconstruction of hippocampal neurons. Scale bar = 50 μm. (B) Dendritic length of hippocampal neurons. (C) Number of branch points in hippocampal neurons. (D) Representative images of dendritic segments. Scale bar =10 μm. (E) Total spine density in the hippocampal neurons. (F) Mushroom spine density in hippocampal neurons. (J) Ultrastructure of neuronal synapses. Scale bar = 500 nm. (H) Synaptic vesicle numbers, synaptic cleft, postsynaptic density (PSD) thickness, and length of the synaptic active zone. (I) mRNA levels of *Syn *and *Psd95.* Data are presented as mean ± SEM. ^*^P< 0.05, ^**^P < 0.01, ^***^P < 0.001. **Fig. S7.** Postnatal IPA supplementation reverses changes in hippocampal neurons of IUGR rats. (A) Representative reconstruction of the hippocampal neurons. Scale bar = 50 μm. (B) Dendritic length of hippocampal neurons. (C) Number of branch points in hippocampal neurons. (D) Representative images of dendritic segments. Scale bar =10 μm. (E) Total spine density in hippocampal neurons. (F) Mushroom spine density in hippocampal neurons. (G) Ultrastructure of neuronal synapses. Scale bar = 500 nm. (H) Synaptic vesicle numbers, synaptic cleft, postsynaptic density (PSD) thickness, and length of the synaptic active zone. (I) mRNA levels of *Syn* and *Psd95*. Dots in panels represent individual samples. Data are presented as mean ± SEM. ^*^P< 0.05, ^**^P < 0.01, ^***^P < 0.001. **Fig. S8.** The methods section is supplemented. (A) The average daily food intake of offspring rats. Dots in panels represent individual samples. (B) Bacterial colonies formed under an anaerobic condition in the feces of SPF rats and pseudo-sterile rats.

**Additional file 2: Table S1.** |List of donors. **Table S2.** |Sequences of primers used for RT-qPCR.

## Data Availability

The 16S RNA gene sequencing data in this study are available in the Sequence Read Archive (SRA) under project number PRJNA857187.

## Abbreviations

ASD: Autism spectrum disorder
IUGR: Intrauterine growth restriction
PCE: Prenatal caffeine exposure
IPA: Indole 3-propionic acid
AHR: Aryl hydrocarbon receptor
PW: Postnatal week
TEM: Transmission electron microscopy
PSD: Postsynaptic density
NF-κB: Nuclear factor kappa-B
P-NF-κB: Phosphorylated-NF-κB
LEfSe: Linear discriminant analysis of effect size
FMT: Fecal microbiota transplantation
GD: Gestational day
PCoA: Principal coordinate analysis

## References

[1] Bougeard C, Picarel-Blanchot F, Schmid R, Campbell R, Buitelaar J (2021). Prevalence of autism spectrum disorder and co-morbidities in children and adolescents: a systematic literature review. Front Psychiatry.

[2] Chiarotti F, Venerosi A (2020). Epidemiology of autism spectrum disorders: a review of worldwide prevalence estimates since 2014. Brain Sci.

[3] Maenner MJ, Shaw KA, Bakian AV, Bilder DA, Durkin MS, Esler A (2021). Prevalence and characteristics of autism spectrum disorder among children aged 8 years - autism and developmental disabilities Monitoring Network, 11 Sites, United States, 2018. Mmwr Surveill Summ.

[4] Gage SH, Munafo MR, Smith GD (2016). Causal inference in developmental origins of health and disease (DOHaD) research. Annu Rev Psychol.

[5] Valsamakis G, Kanaka-Gantenbein C, Malamitsi-Puchner A, Mastorakos G (2006). Causes of intrauterine growth restriction and the postnatal development of the metabolic syndrome. Ann N Y Acad Sci.

[6] Beune IM, Damhuis SE, Ganzevoort W, Hutchinson JC, Khong TY, Mooney EE (2021). Consensus definition of fetal growth restriction in intrauterine fetal death: a delphi procedure. Arch Pathol Lab Med.

[7] Gordijn SJ, Beune IM, Thilaganathan B, Papageorghiou A, Baschat AA, Baker PN (2016). Consensus definition of fetal growth restriction: a delphi procedure. Ultrasound Obstet Gynecol.

[8] Chauhan SP, Beydoun H, Chang E, Sandlin AT, Dahlke JD, Igwe E (2014). Prenatal detection of fetal growth restriction in newborns classified as small for gestational age: correlates and risk of neonatal morbidity. Am J Perinat.

[9] Lee AC, Katz J, Blencowe H, Cousens S, Kozuki N, Vogel JP (2013). National and regional estimates of term and preterm babies born small for gestational age in 138 low-income and middle-income countries in 2010. Lancet Glob Health..

[10] Sacchi C, O'Muircheartaigh J, Batalle D, Counsell SJ, Simonelli A, Cesano M (2021). Neurodevelopmental outcomes following intrauterine growth restriction and very preterm birth. J Pediatr-Us..

[11] Long JY, Guo HL, He X, Hu YH, Xia Y, Cheng R (2021). Caffeine for the pharmacological treatment of apnea of prematurity in the NICU: dose selection conundrum, Therapeutic Drug Monitoring and Genetic Factors. Front Pharmacol.

[12] Maslova E, Bhattacharya S, Lin SW, Michels KB (2010). Caffeine consumption during pregnancy and risk of preterm birth: a meta-analysis. Am J Clin Nutr.

[13] Li S, Wen J, He B, Wang J, Hu X, Liu J (2020). Occurrence of caffeine in the freshwater environment: implications for ecopharmacovigilance. Environ Pollut.

[14] Korekar G, Kumar A, Ugale C (2020). Occurrence, fate, persistence and remediation of caffeine: a review. Environ Sci Pollut Res Int.

[15] Li S, He B, Wang J, Liu J, Hu X (2020). Risks of caffeine residues in the environment: necessity for a targeted ecopharmacovigilance program. Chemosphere.

[16] Group CS (2008). Maternal caffeine intake during pregnancy and risk of fetal growth restriction: a large prospective observational study. BMJ.

[17] Gleason JL, Tekola-Ayele F, Sundaram R, Hinkle SN, Vafai Y, Louis GMB (2021). Association between maternal caffeine consumption and metabolism and neonatal anthropometry a secondary analysis of the NICHD fetal growth studies-singletons. Jama Netw Open.

[18] Soltani S, Salari-Moghaddam A, Saneei P, Askari M, Larijani B, Azadbakht L et al. Maternal caffeine consumption during pregnancy and risk of low birth weight: a dose-response meta-analysis of cohort studies. Crit Rev Food Sci Nutr. 2021:1-10. 10.1080/10408398.2021.1945532. 10.1080/10408398.2021.1945532 34224282

[19] Christensen ZP, Freedman EG, Foxe JJ (2021). Caffeine exposure in utero is associated with structural brain alterations and deleterious neurocognitive outcomes in 9–10 year old children. Neuropharmacology.

[20] Zhang R, Manza P, Volkow ND (2022). Prenatal caffeine exposure: association with neurodevelopmental outcomes in 9- to 11-year-old children. J Child Psychol Psychiatry..

[21] Xu D, Wu Y, Liu F, Liu YS, Shen L, Lei YY (2012). A hypothalamic-pituitary-adrenal axis-associated neuroendocrine metabolic programmed alteration in offspring rats of IUGR induced by prenatal caffeine ingestion. Toxicol Appl Pharmacol..

[22] Xu D, Zhang C, He X, Guo Z, Hu D, Lu J (2018). High expression of hippocampal glutamic acid decarboxylase 67 mediates hypersensitivity of the hypothalamic-pituitary-adrenal axis in response to prenatal caffeine exposure in rats. Toxicol Lett..

[23] Cox LM, Calcagno N, Gauthier C, Madore C, Butovsky O, Weiner HL (2022). The microbiota restrains neurodegenerative microglia in a model of amyotrophic lateral sclerosis. Microbiome..

[24] Seki D, Mayer M, Hausmann B, Pjevac P, Giordano V, Goeral K (2021). Aberrant gut-microbiota-immune-brain axis development in premature neonates with brain damage. Cell Host Microbe.

[25] Bonnechere B, Amin N, van Duijn C (2022). The role of gut microbiota in neuropsychiatric diseases - creation of an atlas-based on quantified evidence. Front Cell Infect Mi.

[26] Yu Y, Zhang B, Ji PF, Zuo ZQ, Huang YX, Wang N (2022). Changes to gut amino acid transporters and microbiome associated with increased E/I ratio in Chd8(+/-) mouse model of ASD-like behavior. Nat Commun.

[27] Li N, Chen HY, Cheng Y, Xu FH, Ruan GC, Ying SH (2021). Fecal microbiota transplantation relieves gastrointestinal and autism symptoms by improving the gut microbiota in an open-label study. Front Cell Infect Mi.

[28] Hamad AF, Alessi-Severini S, Mahmud SM, Brownell M, Kuo IF (2019). Prenatal antibiotics exposure and the risk of autism spectrum disorders: a population-based cohort study. PLoS One.

[29] Mezzelani A, Landini M, Facchiano F, Raggi ME, Villa L, Molteni M (2015). Environment, dysbiosis, immunity and sex-specific susceptibility: a translational hypothesis for regressive autism pathogenesis. Nutr Neurosci..

[30] Zheng H, Xu PT, Jiang QY, Xu QQ, Zheng YF, Yan JJ (2021). Depletion of acetate-producing bacteria from the gut microbiota facilitates cognitive impairment through the gut-brain neural mechanism in diabetic mice. Microbiome.

[31] Xiao WP, Su JB, Gao XJ, Yang H, Weng RY, Ni W (2022). The microbiota-gut-brain axis participates in chronic cerebral hypoperfusion by disrupting the metabolism of short-chain fatty acids. Microbiome.

[32] Hoyles L, Pontifex MG, Rodriguez-Ramiro I, Anis-Alavi MA, Jelane KS, Snelling T (2021). Regulation of blood brain barrier integrity by microbiome-associated methylamines and cognition by trimethylamine N-oxide. Microbiome.

[33] Wikoff WR, Anfora AT, Liu J, Schultz PG, Lesley SA, Peters EC (2009). Metabolomics analysis reveals large effects of gut microflora on mammalian blood metabolites. Proc Natl Acad Sci U S A.

[34] Xiao L, Yan J, Yang T, Zhu J, Li T, Wei H (2021). Fecal microbiome transplantation from children with autism spectrum disorder modulates tryptophan and serotonergic synapse metabolism and induces altered behaviors in germ-free mice. mSystems.

[35] Reinhardt VP, Iosif AM, Libero L, Heath B, Rogers SJ, Ferrer E (2020). Understanding hippocampal development in young children with autism spectrum disorder. J Am Acad Child Adolesc Psychiatry.

[36] Rothhammer V, Borucki DM, Tjon EC, Takenaka MC, Chao CC, Ardura-Fabregat A (2018). Microglial control of astrocytes in response to microbial metabolites. Nature.

[37] Chen GH, Zhang Q, Ai C, Huang SQ, Zhang HZ, Guo XY (2019). Serum metabolic profile characteristics of offspring rats before and after birth caused by prenatal caffeine exposure. Toxicology.

[38] Fombonne E (2009). Epidemiology of pervasive developmental disorders. Pediatr Res.

[39] Behrens TEJ, Muller TH, Whittington JCR, Mark S, Baram AB, Stachenfeld KL (2018). What is a cognitive map? Organizing knowledge for flexible behavior. Neuron..

[40] Cooper RA, Richter FR, Bays PM, Plaisted-Grant KC, Baron-Cohen S, Simons JS (2017). Reduced hippocampal functional connectivity during episodic memory retrieval in autism. Cereb Cortex..

[41] Tao K, Chung M, Watarai A, Huang Z, Wang MY, Okuyama T (2022). Disrupted social memory ensembles in the ventral hippocampus underlie social amnesia in autism-associated Shank3 mutant mice. Mol Psychiatry..

[42] Lituma PJ, Woo E, O'Hara BF, Castillo PE, Sibinga NES, Nandi S. Altered synaptic connectivity and brain function in mice lacking microglial adapter protein Iba1. Proc Natl Acad Sci U S A. 2021;118(46). 10.1073/pnas.2115539118. 10.1073/pnas.2115539118 PMC8609554 34764226

[43] Naik US, Gangadharan C, Abbagani K, Nagalla B, Dasari N, Manna SK (2011). A study of nuclear transcription factor-kappa B in childhood autism. PLoS One.

[44] Qasem H, Al-Ayadhi L, Bjorklund G, Chirumbolo S, El-Ansary A (2018). Impaired lipid metabolism markers to assess the risk of neuroinflammation in autism spectrum disorder. Metab Brain Dis..

[45] Dominguez-Acost O, Vega L, Estrada-Muniz E, Rodriguez MS, Gonzalez FJ, Elizondo G (2018). Activation of aryl hydrocarbon receptor regulates the LPS/IFN gamma-induced inflammatory response by inducing ubiquitin-proteosomal and lysosomal degradation of RelA/p65. Biochemical Pharmacology..

[46] Serger E, Luengo-Gutierrez L, Chadwick JS, Kong G, Zhou L, Crawford G (2022). The gut metabolite indole-3 propionate promotes nerve regeneration and repair. Nature..

[47] Dodd D, Spitzer MH, Van Treuren W, Merrill BD, Hryckowian AJ, Higginbottom SK (2017). A gut bacterial pathway metabolizes aromatic amino acids into nine circulating metabolites. Nature.

[48] Martin-Estal I, de la Garza RG, Castilla-Cortazar I. Intrauterine growth retardation (IUGR) as a novel condition of insulin-like growth factor-1 (IGF-1) deficiency (vol 170, pg 1, 2016). Rev Physiol Bioch P. 2016;170:129-. doi:10.1007/112_2016_1. 10.1007/112_2016_1 27012750

[49] Shrivastava D, Master A (2020). Fetal growth restriction. J Obstet Gynaecol India.

[50] Chen LW, Wu Y, Neelakantan N, Chong MFF, Pan A, van Dam RM (2014). Maternal caffeine intake during pregnancy is associated with risk of low birth weight: a systematic review and dose-response meta-analysis. Bmc Med.

[51] Greenwood DC, Thatcher NJ, Ye J, Garrard L, Keogh G, King LG (2014). Caffeine intake during pregnancy and adverse birth outcomes: a systematic review and dose-response meta-analysis. Eur J Epidemiol.

[52] Ge CY, Xu D, Yu PX, Fang M, Guo JJ, Xu D (2021). P-gp expression inhibition mediates placental glucocorticoid barrier opening and fetal weight loss. BMC Med.

[53] He H, Luo H, Liu L, Shangguan Y, Xie X, Wen Y (2021). Prenatal caffeine exposure caused H-type blood vessel-related long bone dysplasia via miR375/CTGF signaling. FASEB J.

[54] He Z, Zhang J, Chen G, Cao J, Chen Y, Ai C (2021). H19/let-7 axis mediates caffeine exposure during pregnancy induced adrenal dysfunction and its multi-generation inheritance. Sci Total Environ.

[55] Lee IC, Wang YH, Chiou JY, Wei JC (2022). Perinatal factors in newborn are insidious risk factors for childhood autism spectrum disorders: a population-based study. J Autism Dev Disord.

[56] Schendel D, Bhasin TK (2008). Birth weight and gestational age characteristics of children with autism, including a comparison with other developmental disabilities. Pediatrics.

[57] Talmi Z, Mankuta D, Raz R (2020). Birth weight and autism spectrum disorder: a population-based nested case-control study. Autism Res.

[58] Anderson PJ, de Miranda DM, Albuquerque MR, Indredavik MS, Evensen KAI, Van Lieshout R (2021). Psychiatric disorders in individuals born very preterm / very low-birth weight: an individual participant data (IPD) meta-analysis. EClinicalMedicine.

[59] Lee PS, Yerys BE, Della Rosa A, Foss-Feig J, Barnes KA, James JD (2009). Functional connectivity of the inferior frontal cortex changes with age in children with autism spectrum disorders: a fcMRI study of response inhibition. Cereb Cortex..

[60] Baron-Cohen S, Ring HA, Bullmore ET, Wheelwright S, Ashwin C, Williams SC (2000). The amygdala theory of autism. Neurosci Biobehav Rev.

[61] Bertoni A, Schaller F, Tyzio R, Gaillard S, Santini F, Xolin M (2021). Oxytocin administration in neonates shapes hippocampal circuitry and restores social behavior in a mouse model of autism. Mol Psychiatr.

[62] Banker SM, Gu X, Schiller D, Foss-Feig JH (2021). Hippocampal contributions to social and cognitive deficits in autism spectrum disorder. Trends Neurosci.

[63] Braden BB, Dassel KB, Bimonte-Nelson HA, O'Rourke HP, Connor DJ, Moorhous S (2017). Sex and post-menopause hormone therapy effects on hippocampal volume and verbal memory. Aging Neuropsychol C.

[64] Hitti FL, Siegelbaum SA (2014). The hippocampal CA2 region is essential for social memory. Nature.

[65] Kim HJ, Cho MH, Shim WH, Kim JK, Jeon EY, Kim DH (2017). Deficient autophagy in microglia impairs synaptic pruning and causes social behavioral defects. Mol Psychiatry.

[66] Sacai H, Sakoori K, Konno K, Nagahama K, Suzuki H, Watanabe T (2020). Autism spectrum disorder-like behavior caused by reduced excitatory synaptic transmission in pyramidal neurons of mouse prefrontal cortex. Nat Commun.

[67] Young AM, Campbell E, Lynch S, Suckling J, Powis SJ (2011). Aberrant NF-kappaB expression in autism spectrum condition: a mechanism for neuroinflammation. Front Psychiatry.

[68] de la Parra J, Cuartero MI, Perez-Ruiz A, Garcia-Culebras A, Martin R, Sanchez-Prieto J et al. AhR deletion promotes aberrant morphogenesis and synaptic activity of adult-generated granule neurons and impairs hippocampus-dependent memory. eNeuro. 2018;5(4). 10.1523/ENEURO.0370-17.2018. 10.1523/ENEURO.0370-17.2018 PMC6140122 30225360

[69] Fujisawa TX, Nishitani S, Iwanaga R, Matsuzaki J, Kawasaki C, Tochigi M (2016). Association of aryl hydrocarbon receptor-related gene variants with the severity of autism spectrum disorders. Front Psychiatry.

[70] Rothhammer V, Quintana FJ (2019). The aryl hydrocarbon receptor: an environmental sensor integrating immune responses in health and disease. Nat Rev Immunol.

[71] Tartaglione AM, Villani A, Ajmone-Cat MA, Minghetti L, Ricceri L, Pazienza V (2022). Maternal immune activation induces autism-like changes in behavior, neuroinflammatory profile and gut microbiota in mouse offspring of both sexes. Transl Psychiat.

[72] Yap CX, Henders AK, Alvares GA, Wood DLA, Krause L, Tyson GW (2021). Autism-related dietary preferences mediate autism-gut microbiome associations. Cell.

[73] Lord C, Elsabbagh M, Baird G, Veenstra-Vanderweele J (2018). Autism spectrum disorder. Lancet.

[74] Roswall J, Olsson LM, Kovatcheva-Datchary P, Nilsson S, Tremaroli V, Simon MC (2021). Developmental trajectory of the healthy human gut microbiota during the first 5 years of life. Cell Host Microbe.

[75] Lou M, Cao A, Jin C, Mi K, Xiong X, Zeng Z (2022). Deviated and early unsustainable stunted development of gut microbiota in children with autism spectrum disorder. Gut.

[76] Sharon G, Cruz NJ, Kang DW, Gandal MJ, Wang B, Kim YM (2019). Human gut microbiota from autism spectrum disorder promote behavioral symptoms in mice. Cell.

[77] Liu X, Li X, Xia B, Jin X, Zou Q, Zeng Z (2021). High-fiber diet mitigates maternal obesity-induced cognitive and social dysfunction in the offspring via gut-brain axis. Cell Metab.

[78] Tamburini S, Shen N, Wu HC, Clemente JC (2016). The microbiome in early life: implications for health outcomes. Nat Med.

[79] Codagnone MG, Spichak S, O'Mahony SM, O'Leary OF, Clarke G, Stanton C (2019). Programming bugs: microbiota and the developmental origins of brain health and disease. Biol Psychiat.

[80] Iovene MR, Bombace F, Maresca R, Sapone A, Iardino P, Picardi A (2017). Intestinal dysbiosis and yeast isolation in stool of subjects with autism spectrum disorders. Mycopathologia.

[81] Hwang IK, Yoo KY, Li H, Park OK, Lee CH, Choi JH (2009). Indole-3-propionic acid attenuates neuronal damage and oxidative stress in the ischemic hippocampus. J Neurosci Res.

[82] Roager HM, Licht TR (2018). Microbial tryptophan catabolites in health and disease. Nat Commun.

[83] Hu S, Xia L, Luo H, Xu Y, Yu H, Xu D (2019). Prenatal caffeine exposure increases the susceptibility to non-alcoholic fatty liver disease in female offspring rats via activation of GR-C/EBPalpha-SIRT1 pathway. Toxicology.

[84] Wilson-Ching M, Pascoe L, Doyle LW, Anderson PJ (2014). Effects of correcting for prematurity on cognitive test scores in childhood. J Paediatr Child Health.

[85] Wang JJ, Zhang ZY, Chen OU (2022). What is the impact of birth weight corrected for gestational age on later onset asthma: a meta-analysis. Allergy Asthma Cl Im.

[86] Gould JF, Fuss BG, Roberts RM, Collins CT, Makrides M (2021). Consequences of using chronological age versus corrected age when testing cognitive and motor development in infancy and intelligence quotient at school age for children born preterm. Plos One.

[87] Lv Q, Wang K, Qiao S, Yang L, Xin Y, Dai Y (2018). Norisoboldine, a natural AhR agonist, promotes Treg differentiation and attenuates colitis via targeting glycolysis and subsequent NAD(+)/SIRT1/SUV39H1/H3K9me3 signaling pathway. Cell Death Dis.

[88] Yan J, Tung HC, Li S, Niu Y, Garbacz WG, Lu P (2019). Aryl hydrocarbon receptor signaling prevents activation of hepatic stellate cells and liver fibrogenesis in mice. Gastroenterology.

[89] Zhao ZH, Xin FZ, Xue Y, Hu Z, Han Y, Ma F (2019). Indole-3-propionic acid inhibits gut dysbiosis and endotoxin leakage to attenuate steatohepatitis in rats. Exp Mol Med.

[90] Zhao ZH, Xin FZ, Xue YQ, Hu ZM, Han YM, Ma FG (2019). Indole-3-propionic acid inhibits gut dysbiosis and endotoxin leakage to attenuate steatohepatitis in rats. Exp Mol Med.

[91] Wu WL, Adame MD, Liou CW, Barlow JT, Lai TT, Sharon G (2021). Microbiota regulate social behaviour via stress response neurons in the brain. Nature.

[92] Wang C, Liu H, Li K, Wu ZZ, Wu C, Yu JY (2020). Tactile modulation of memory and anxiety requires dentate granule cells along the dorsoventral axis. Nat Commun.

[93] Jiang T, Dai SY, Yi YW, Liu Y, Zhang S, Luo MC (2020). The combination of hprt and gapdh is the best compound reference genes in the fetal rat hippocampus. Dev Neurobiol.

