# Supplementary figures and images for: Microbiota-indole 3-propionic acid-brain axis mediates abnormal synaptic pruning of hippocampal microglia and susceptibility to ASD in IUGR offspring
Source: Microbiome. 2023 Nov 7;11:245. doi: 10.1186/s40168-023-01656-1 (PMC10629055; doi:10.1186/s40168-023-01656-1)

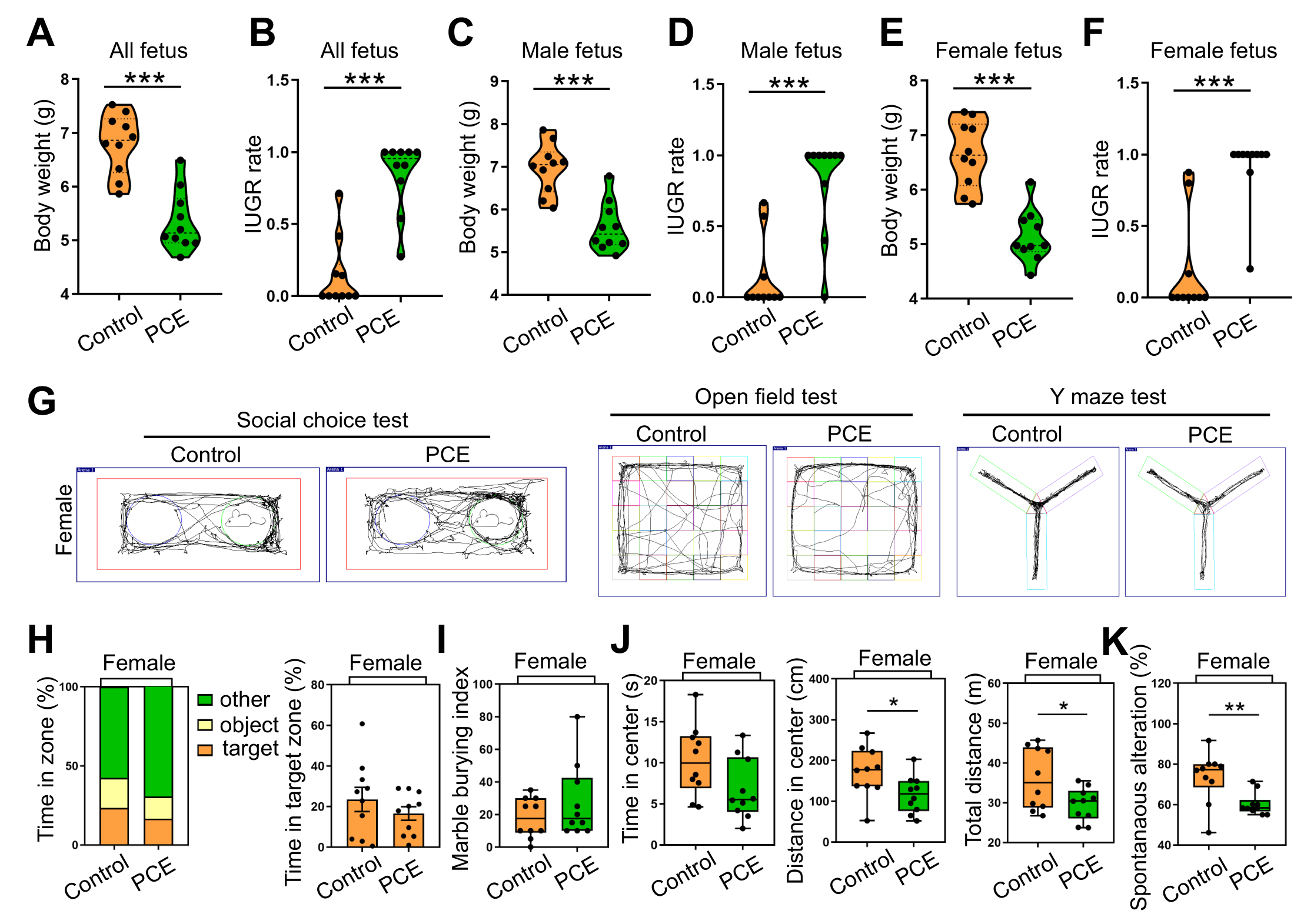

Supplement: Supplementary file 2 — Additional file 1: Fig. S1. PCE-induced IUGR female rats did not show typical ASD-like manifestations. (A, C) Fetal body weight on the first day after birth. (B, D) IUGR rate. (E) An illustrative example of travel pathways of female rats in social choice test, open field test, and Y maze test. (F) Time of female rats spent in each zone in social choice test. (G) Marble burying index in marble burying test. (H) Time spent in the center, distance traveled in the center, and total distance traveled in open field test. (I) Spontaneous alteration rate in Y maze test. Dots in panels represent individual samples. Data are presented as mean ± SEM. *P < 0.05, ***P< 0.01. Fig. S2. AHR/NF-κB signaling is involved in ASD. (A) The top enriched pathways of differentially expressed genes by Clusterprofiler. (B) Gene set enrichment analysis (GSEA) plots shows NF-κB signaling pathway. Fig. S3. AHR intervention induces abnormalities of neurons and activation of microglia in the co-cultured primary hippocampal microglia and neurons in vitro. (A) Representative reconstruction of hippocampal neurons. Scale bar = 50 μm. (B) Dendritic length of hippocampal neurons. (C) Number of branch points in hippocampal neurons. (D) Dose-effect: cell viability after treated with different concentrations of CH-223191 (0, 5, 10, 20, 40 or 80 μM) for 2 h. (E) Time-effect: cell viability after treated with 10 μM CH-223191 at different time (0, 0.5, 1, 2, 4, and 8 h). (F) Protein levels of AHR and P-NF-κB. (G) mRNA levels of Ahr. (H) Morphology of primary microglia under the light microscope. Scale bar = 50 μm. (I) Morphology of primary microglia under the fluorescence microscope. Iba1 staining (green) and nuclear staining (DIPA, blue). The arrows point at activated microglia. Scale bar = 50 μm. (J) Microglia activation rate. Dots in panels represent individual samples. Data are presented as mean ± SEM. *P < 0.05, **P< 0.01, ***P < 0.001. Fig. S4. Correlation analysis and gut microbiota signatures of donor [file 40168_2023_1656_MOESM1_ESM.zip › Supplementary Fig. S1.tif]

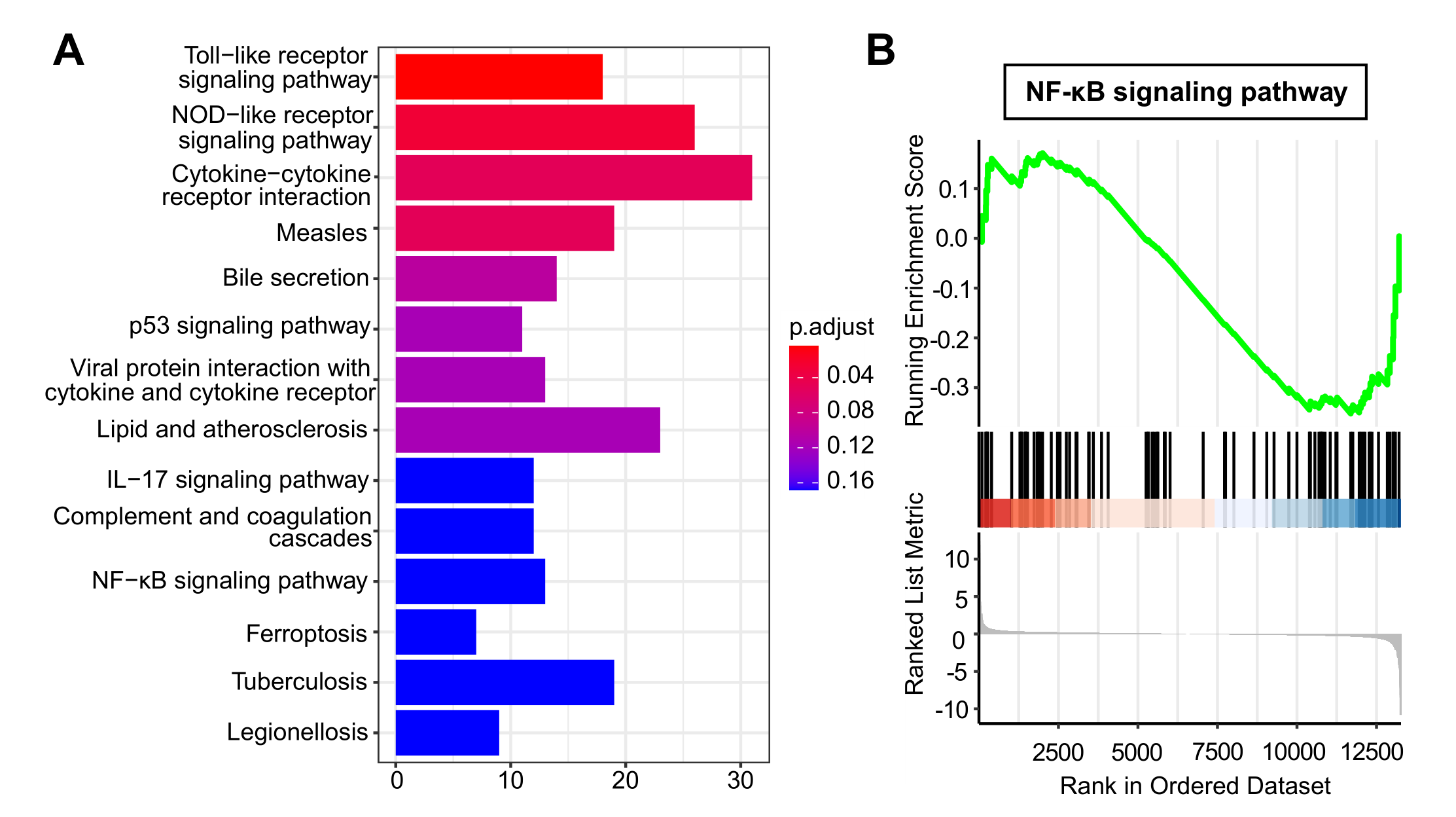

Supplement: Supplementary file 2 — Additional file 1: Fig. S1. PCE-induced IUGR female rats did not show typical ASD-like manifestations. (A, C) Fetal body weight on the first day after birth. (B, D) IUGR rate. (E) An illustrative example of travel pathways of female rats in social choice test, open field test, and Y maze test. (F) Time of female rats spent in each zone in social choice test. (G) Marble burying index in marble burying test. (H) Time spent in the center, distance traveled in the center, and total distance traveled in open field test. (I) Spontaneous alteration rate in Y maze test. Dots in panels represent individual samples. Data are presented as mean ± SEM. *P < 0.05, ***P< 0.01. Fig. S2. AHR/NF-κB signaling is involved in ASD. (A) The top enriched pathways of differentially expressed genes by Clusterprofiler. (B) Gene set enrichment analysis (GSEA) plots shows NF-κB signaling pathway. Fig. S3. AHR intervention induces abnormalities of neurons and activation of microglia in the co-cultured primary hippocampal microglia and neurons in vitro. (A) Representative reconstruction of hippocampal neurons. Scale bar = 50 μm. (B) Dendritic length of hippocampal neurons. (C) Number of branch points in hippocampal neurons. (D) Dose-effect: cell viability after treated with different concentrations of CH-223191 (0, 5, 10, 20, 40 or 80 μM) for 2 h. (E) Time-effect: cell viability after treated with 10 μM CH-223191 at different time (0, 0.5, 1, 2, 4, and 8 h). (F) Protein levels of AHR and P-NF-κB. (G) mRNA levels of Ahr. (H) Morphology of primary microglia under the light microscope. Scale bar = 50 μm. (I) Morphology of primary microglia under the fluorescence microscope. Iba1 staining (green) and nuclear staining (DIPA, blue). The arrows point at activated microglia. Scale bar = 50 μm. (J) Microglia activation rate. Dots in panels represent individual samples. Data are presented as mean ± SEM. *P < 0.05, **P< 0.01, ***P < 0.001. Fig. S4. Correlation analysis and gut microbiota signatures of donor [file 40168_2023_1656_MOESM1_ESM.zip › Supplementary Fig. S2.tif]

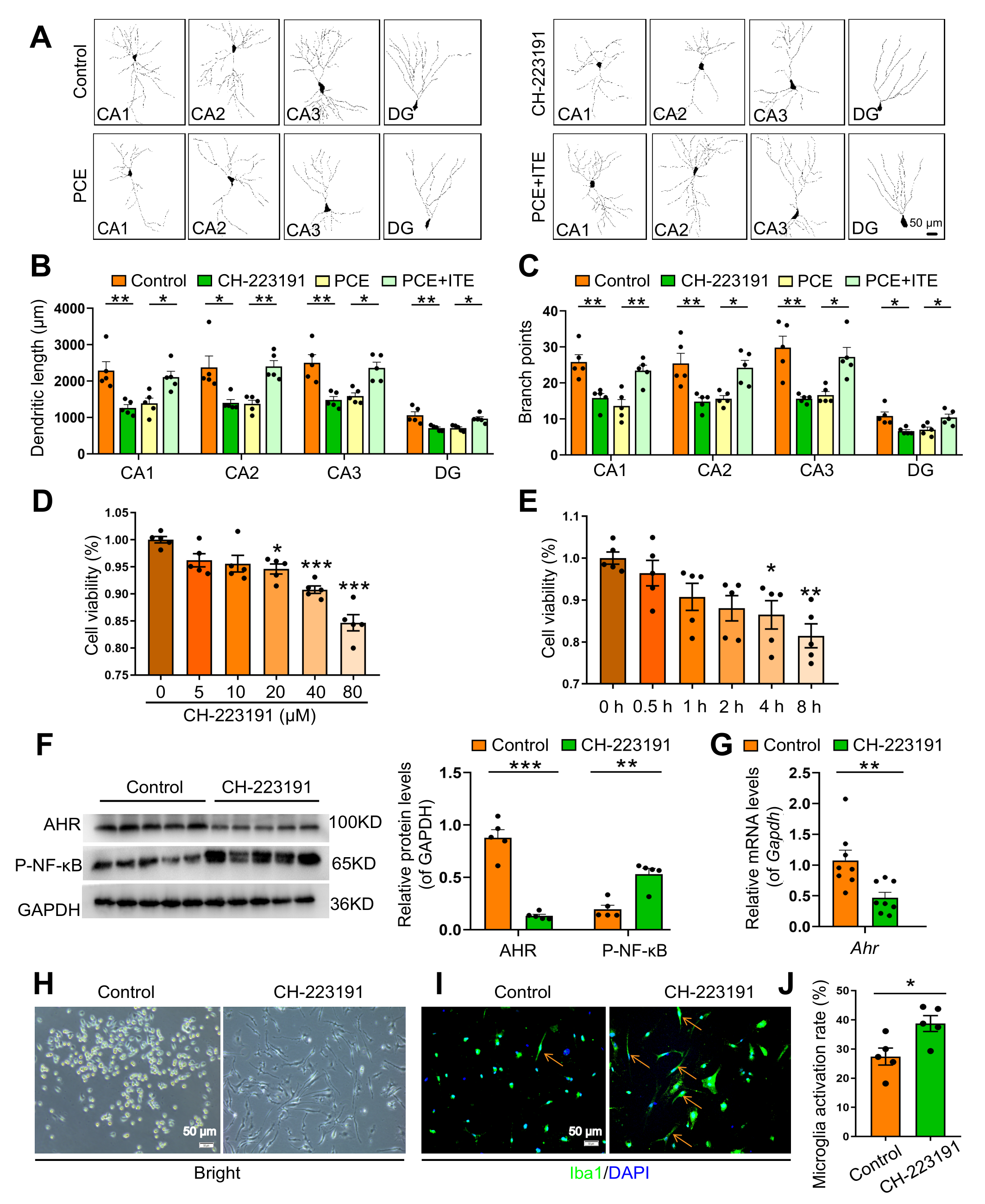

Supplement: Supplementary file 2 — Additional file 1: Fig. S1. PCE-induced IUGR female rats did not show typical ASD-like manifestations. (A, C) Fetal body weight on the first day after birth. (B, D) IUGR rate. (E) An illustrative example of travel pathways of female rats in social choice test, open field test, and Y maze test. (F) Time of female rats spent in each zone in social choice test. (G) Marble burying index in marble burying test. (H) Time spent in the center, distance traveled in the center, and total distance traveled in open field test. (I) Spontaneous alteration rate in Y maze test. Dots in panels represent individual samples. Data are presented as mean ± SEM. *P < 0.05, ***P< 0.01. Fig. S2. AHR/NF-κB signaling is involved in ASD. (A) The top enriched pathways of differentially expressed genes by Clusterprofiler. (B) Gene set enrichment analysis (GSEA) plots shows NF-κB signaling pathway. Fig. S3. AHR intervention induces abnormalities of neurons and activation of microglia in the co-cultured primary hippocampal microglia and neurons in vitro. (A) Representative reconstruction of hippocampal neurons. Scale bar = 50 μm. (B) Dendritic length of hippocampal neurons. (C) Number of branch points in hippocampal neurons. (D) Dose-effect: cell viability after treated with different concentrations of CH-223191 (0, 5, 10, 20, 40 or 80 μM) for 2 h. (E) Time-effect: cell viability after treated with 10 μM CH-223191 at different time (0, 0.5, 1, 2, 4, and 8 h). (F) Protein levels of AHR and P-NF-κB. (G) mRNA levels of Ahr. (H) Morphology of primary microglia under the light microscope. Scale bar = 50 μm. (I) Morphology of primary microglia under the fluorescence microscope. Iba1 staining (green) and nuclear staining (DIPA, blue). The arrows point at activated microglia. Scale bar = 50 μm. (J) Microglia activation rate. Dots in panels represent individual samples. Data are presented as mean ± SEM. *P < 0.05, **P< 0.01, ***P < 0.001. Fig. S4. Correlation analysis and gut microbiota signatures of donor [file 40168_2023_1656_MOESM1_ESM.zip › Supplementary Fig. S3.tif]

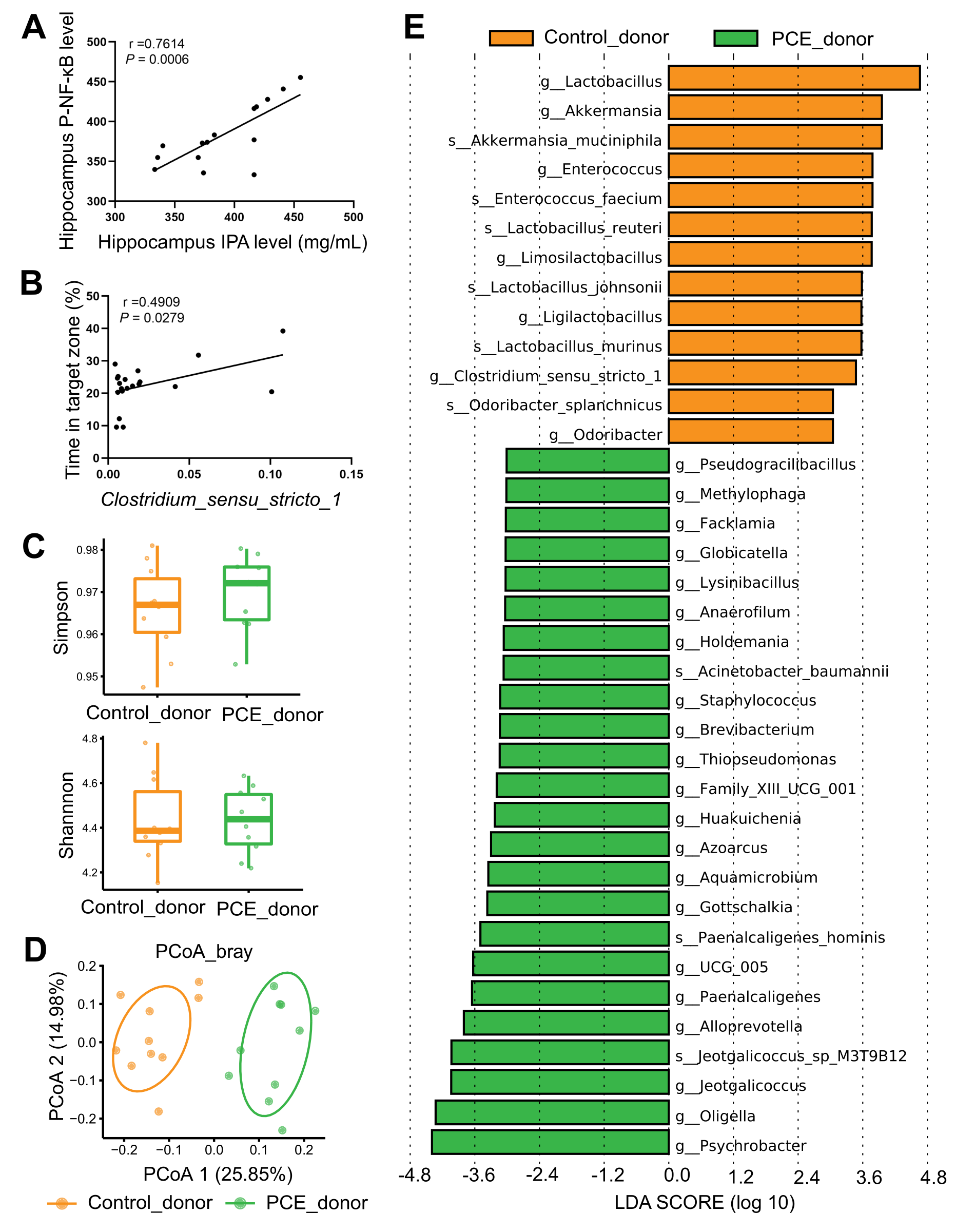

Supplement: Supplementary file 2 — Additional file 1: Fig. S1. PCE-induced IUGR female rats did not show typical ASD-like manifestations. (A, C) Fetal body weight on the first day after birth. (B, D) IUGR rate. (E) An illustrative example of travel pathways of female rats in social choice test, open field test, and Y maze test. (F) Time of female rats spent in each zone in social choice test. (G) Marble burying index in marble burying test. (H) Time spent in the center, distance traveled in the center, and total distance traveled in open field test. (I) Spontaneous alteration rate in Y maze test. Dots in panels represent individual samples. Data are presented as mean ± SEM. *P < 0.05, ***P< 0.01. Fig. S2. AHR/NF-κB signaling is involved in ASD. (A) The top enriched pathways of differentially expressed genes by Clusterprofiler. (B) Gene set enrichment analysis (GSEA) plots shows NF-κB signaling pathway. Fig. S3. AHR intervention induces abnormalities of neurons and activation of microglia in the co-cultured primary hippocampal microglia and neurons in vitro. (A) Representative reconstruction of hippocampal neurons. Scale bar = 50 μm. (B) Dendritic length of hippocampal neurons. (C) Number of branch points in hippocampal neurons. (D) Dose-effect: cell viability after treated with different concentrations of CH-223191 (0, 5, 10, 20, 40 or 80 μM) for 2 h. (E) Time-effect: cell viability after treated with 10 μM CH-223191 at different time (0, 0.5, 1, 2, 4, and 8 h). (F) Protein levels of AHR and P-NF-κB. (G) mRNA levels of Ahr. (H) Morphology of primary microglia under the light microscope. Scale bar = 50 μm. (I) Morphology of primary microglia under the fluorescence microscope. Iba1 staining (green) and nuclear staining (DIPA, blue). The arrows point at activated microglia. Scale bar = 50 μm. (J) Microglia activation rate. Dots in panels represent individual samples. Data are presented as mean ± SEM. *P < 0.05, **P< 0.01, ***P < 0.001. Fig. S4. Correlation analysis and gut microbiota signatures of donor [file 40168_2023_1656_MOESM1_ESM.zip › Supplementary Fig. S4.tif]

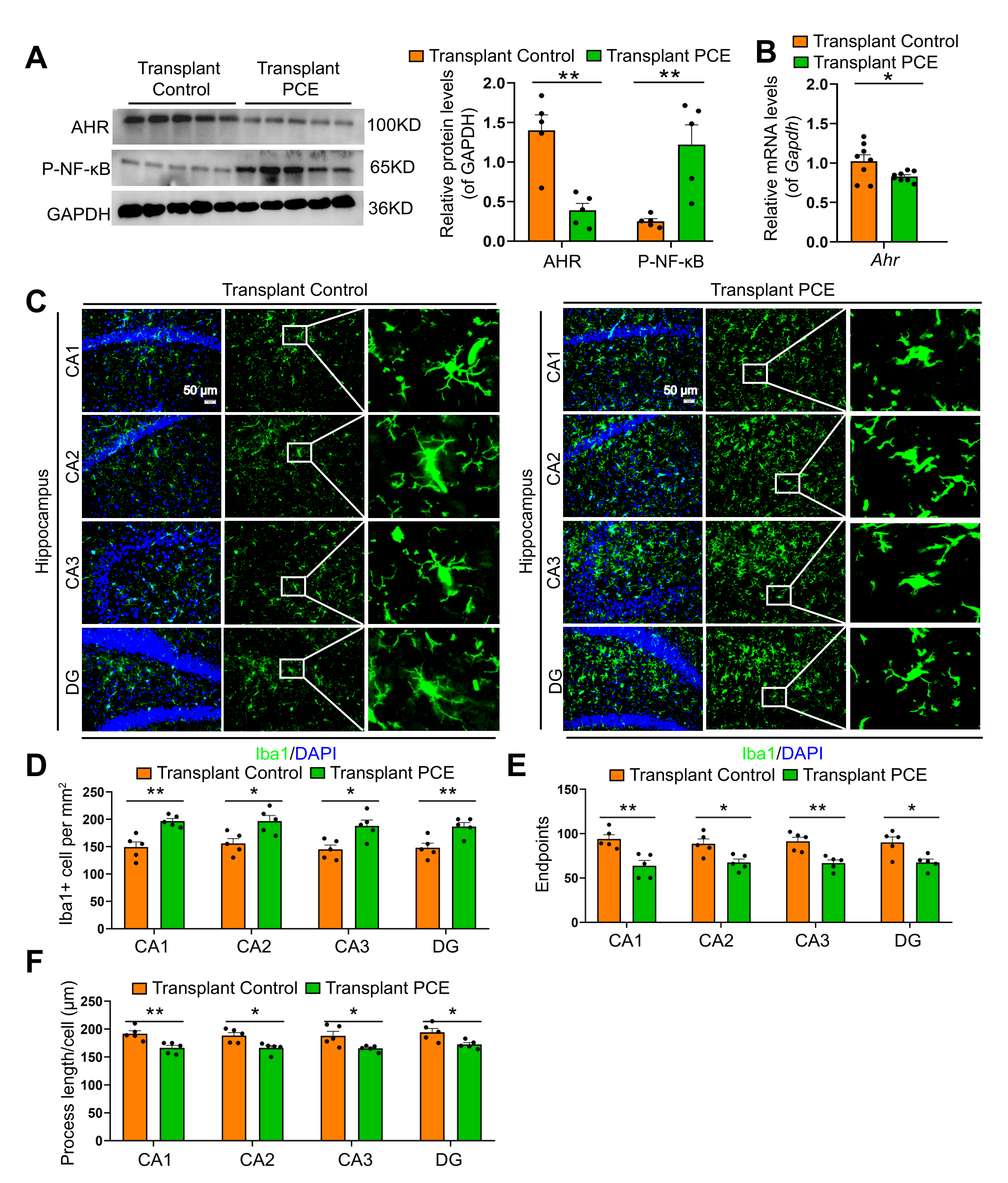

Supplement: Supplementary file 2 — Additional file 1: Fig. S1. PCE-induced IUGR female rats did not show typical ASD-like manifestations. (A, C) Fetal body weight on the first day after birth. (B, D) IUGR rate. (E) An illustrative example of travel pathways of female rats in social choice test, open field test, and Y maze test. (F) Time of female rats spent in each zone in social choice test. (G) Marble burying index in marble burying test. (H) Time spent in the center, distance traveled in the center, and total distance traveled in open field test. (I) Spontaneous alteration rate in Y maze test. Dots in panels represent individual samples. Data are presented as mean ± SEM. *P < 0.05, ***P< 0.01. Fig. S2. AHR/NF-κB signaling is involved in ASD. (A) The top enriched pathways of differentially expressed genes by Clusterprofiler. (B) Gene set enrichment analysis (GSEA) plots shows NF-κB signaling pathway. Fig. S3. AHR intervention induces abnormalities of neurons and activation of microglia in the co-cultured primary hippocampal microglia and neurons in vitro. (A) Representative reconstruction of hippocampal neurons. Scale bar = 50 μm. (B) Dendritic length of hippocampal neurons. (C) Number of branch points in hippocampal neurons. (D) Dose-effect: cell viability after treated with different concentrations of CH-223191 (0, 5, 10, 20, 40 or 80 μM) for 2 h. (E) Time-effect: cell viability after treated with 10 μM CH-223191 at different time (0, 0.5, 1, 2, 4, and 8 h). (F) Protein levels of AHR and P-NF-κB. (G) mRNA levels of Ahr. (H) Morphology of primary microglia under the light microscope. Scale bar = 50 μm. (I) Morphology of primary microglia under the fluorescence microscope. Iba1 staining (green) and nuclear staining (DIPA, blue). The arrows point at activated microglia. Scale bar = 50 μm. (J) Microglia activation rate. Dots in panels represent individual samples. Data are presented as mean ± SEM. *P < 0.05, **P< 0.01, ***P < 0.001. Fig. S4. Correlation analysis and gut microbiota signatures of donor [file 40168_2023_1656_MOESM1_ESM.zip › Supplementary Fig. S5.tif]

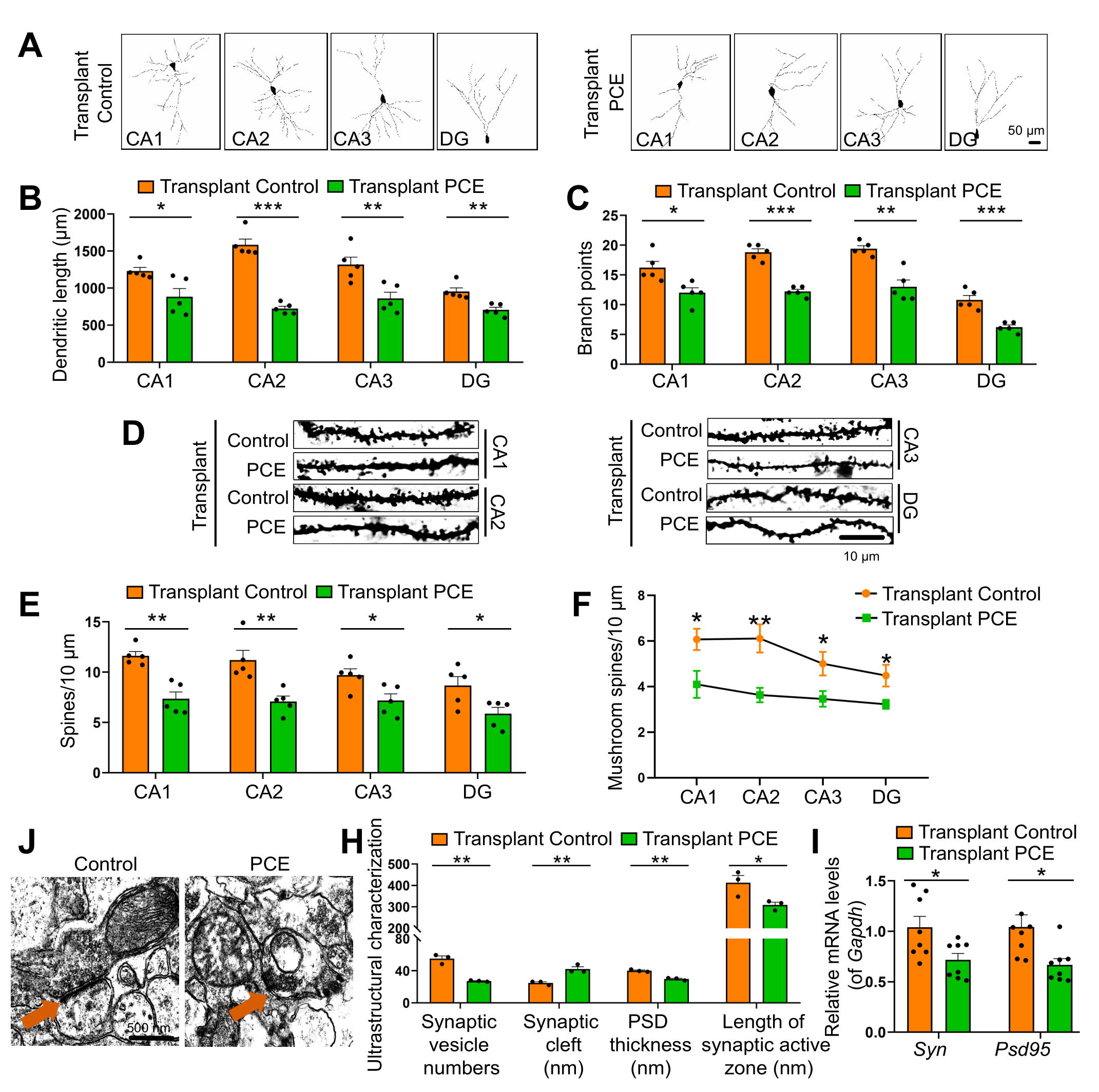

Supplement: Supplementary file 2 — Additional file 1: Fig. S1. PCE-induced IUGR female rats did not show typical ASD-like manifestations. (A, C) Fetal body weight on the first day after birth. (B, D) IUGR rate. (E) An illustrative example of travel pathways of female rats in social choice test, open field test, and Y maze test. (F) Time of female rats spent in each zone in social choice test. (G) Marble burying index in marble burying test. (H) Time spent in the center, distance traveled in the center, and total distance traveled in open field test. (I) Spontaneous alteration rate in Y maze test. Dots in panels represent individual samples. Data are presented as mean ± SEM. *P < 0.05, ***P< 0.01. Fig. S2. AHR/NF-κB signaling is involved in ASD. (A) The top enriched pathways of differentially expressed genes by Clusterprofiler. (B) Gene set enrichment analysis (GSEA) plots shows NF-κB signaling pathway. Fig. S3. AHR intervention induces abnormalities of neurons and activation of microglia in the co-cultured primary hippocampal microglia and neurons in vitro. (A) Representative reconstruction of hippocampal neurons. Scale bar = 50 μm. (B) Dendritic length of hippocampal neurons. (C) Number of branch points in hippocampal neurons. (D) Dose-effect: cell viability after treated with different concentrations of CH-223191 (0, 5, 10, 20, 40 or 80 μM) for 2 h. (E) Time-effect: cell viability after treated with 10 μM CH-223191 at different time (0, 0.5, 1, 2, 4, and 8 h). (F) Protein levels of AHR and P-NF-κB. (G) mRNA levels of Ahr. (H) Morphology of primary microglia under the light microscope. Scale bar = 50 μm. (I) Morphology of primary microglia under the fluorescence microscope. Iba1 staining (green) and nuclear staining (DIPA, blue). The arrows point at activated microglia. Scale bar = 50 μm. (J) Microglia activation rate. Dots in panels represent individual samples. Data are presented as mean ± SEM. *P < 0.05, **P< 0.01, ***P < 0.001. Fig. S4. Correlation analysis and gut microbiota signatures of donor [file 40168_2023_1656_MOESM1_ESM.zip › Supplementary Fig. S6.tif]

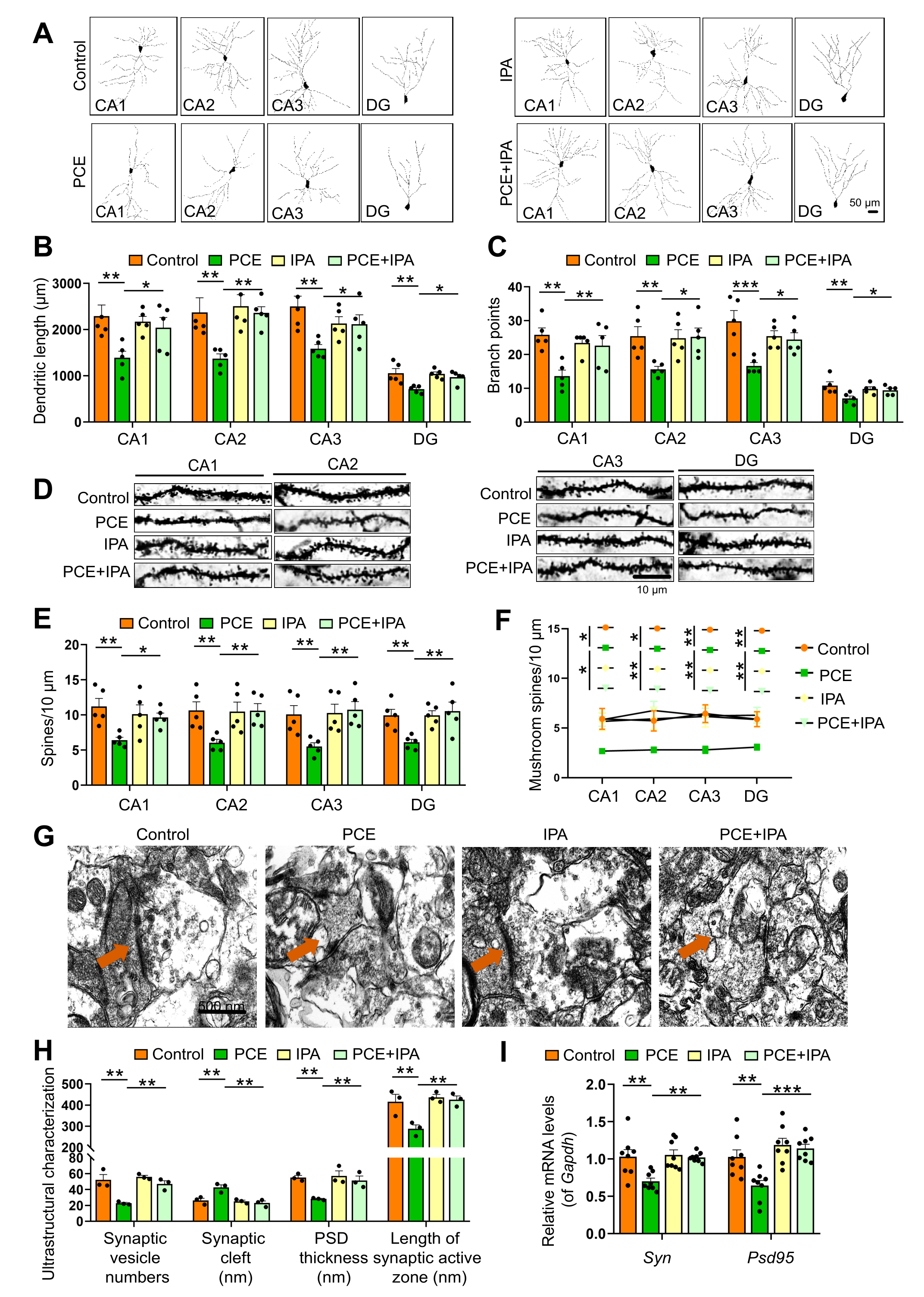

Supplement: Supplementary file 2 — Additional file 1: Fig. S1. PCE-induced IUGR female rats did not show typical ASD-like manifestations. (A, C) Fetal body weight on the first day after birth. (B, D) IUGR rate. (E) An illustrative example of travel pathways of female rats in social choice test, open field test, and Y maze test. (F) Time of female rats spent in each zone in social choice test. (G) Marble burying index in marble burying test. (H) Time spent in the center, distance traveled in the center, and total distance traveled in open field test. (I) Spontaneous alteration rate in Y maze test. Dots in panels represent individual samples. Data are presented as mean ± SEM. *P < 0.05, ***P< 0.01. Fig. S2. AHR/NF-κB signaling is involved in ASD. (A) The top enriched pathways of differentially expressed genes by Clusterprofiler. (B) Gene set enrichment analysis (GSEA) plots shows NF-κB signaling pathway. Fig. S3. AHR intervention induces abnormalities of neurons and activation of microglia in the co-cultured primary hippocampal microglia and neurons in vitro. (A) Representative reconstruction of hippocampal neurons. Scale bar = 50 μm. (B) Dendritic length of hippocampal neurons. (C) Number of branch points in hippocampal neurons. (D) Dose-effect: cell viability after treated with different concentrations of CH-223191 (0, 5, 10, 20, 40 or 80 μM) for 2 h. (E) Time-effect: cell viability after treated with 10 μM CH-223191 at different time (0, 0.5, 1, 2, 4, and 8 h). (F) Protein levels of AHR and P-NF-κB. (G) mRNA levels of Ahr. (H) Morphology of primary microglia under the light microscope. Scale bar = 50 μm. (I) Morphology of primary microglia under the fluorescence microscope. Iba1 staining (green) and nuclear staining (DIPA, blue). The arrows point at activated microglia. Scale bar = 50 μm. (J) Microglia activation rate. Dots in panels represent individual samples. Data are presented as mean ± SEM. *P < 0.05, **P< 0.01, ***P < 0.001. Fig. S4. Correlation analysis and gut microbiota signatures of donor [file 40168_2023_1656_MOESM1_ESM.zip › Supplementary Fig. S7.tif]

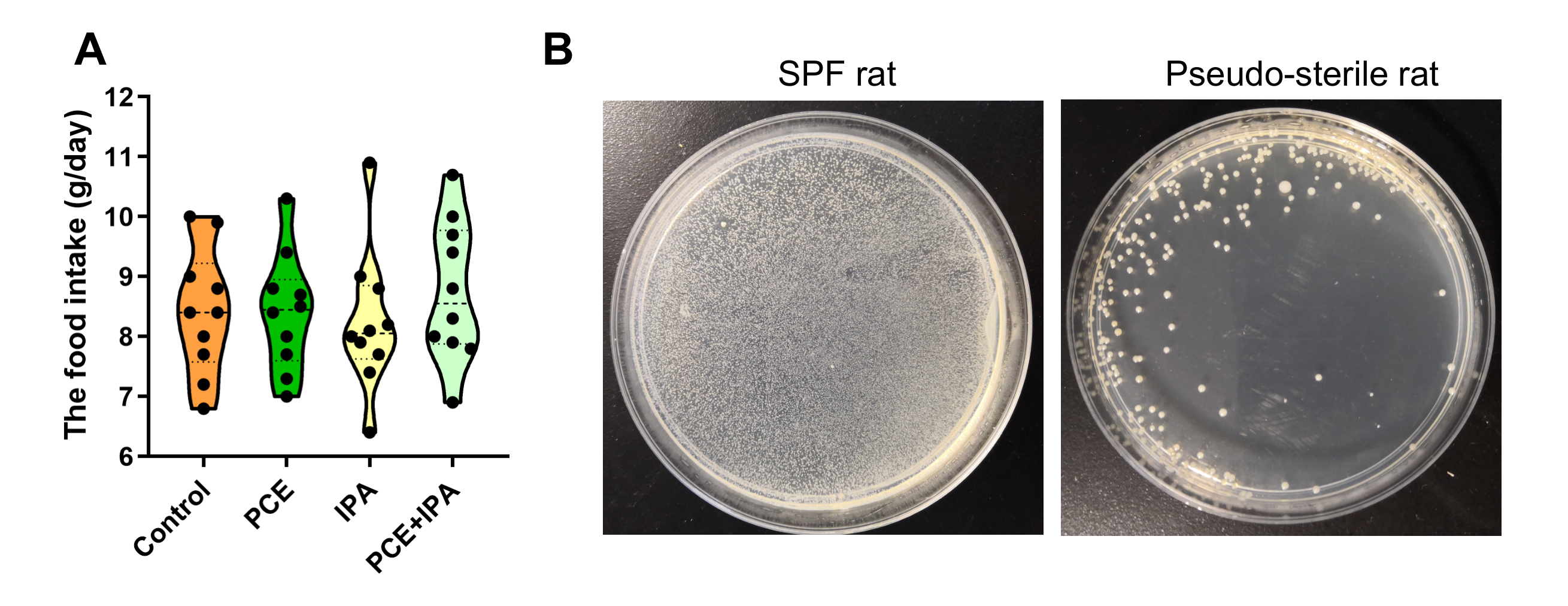

Supplement: Supplementary file 2 — Additional file 1: Fig. S1. PCE-induced IUGR female rats did not show typical ASD-like manifestations. (A, C) Fetal body weight on the first day after birth. (B, D) IUGR rate. (E) An illustrative example of travel pathways of female rats in social choice test, open field test, and Y maze test. (F) Time of female rats spent in each zone in social choice test. (G) Marble burying index in marble burying test. (H) Time spent in the center, distance traveled in the center, and total distance traveled in open field test. (I) Spontaneous alteration rate in Y maze test. Dots in panels represent individual samples. Data are presented as mean ± SEM. *P < 0.05, ***P< 0.01. Fig. S2. AHR/NF-κB signaling is involved in ASD. (A) The top enriched pathways of differentially expressed genes by Clusterprofiler. (B) Gene set enrichment analysis (GSEA) plots shows NF-κB signaling pathway. Fig. S3. AHR intervention induces abnormalities of neurons and activation of microglia in the co-cultured primary hippocampal microglia and neurons in vitro. (A) Representative reconstruction of hippocampal neurons. Scale bar = 50 μm. (B) Dendritic length of hippocampal neurons. (C) Number of branch points in hippocampal neurons. (D) Dose-effect: cell viability after treated with different concentrations of CH-223191 (0, 5, 10, 20, 40 or 80 μM) for 2 h. (E) Time-effect: cell viability after treated with 10 μM CH-223191 at different time (0, 0.5, 1, 2, 4, and 8 h). (F) Protein levels of AHR and P-NF-κB. (G) mRNA levels of Ahr. (H) Morphology of primary microglia under the light microscope. Scale bar = 50 μm. (I) Morphology of primary microglia under the fluorescence microscope. Iba1 staining (green) and nuclear staining (DIPA, blue). The arrows point at activated microglia. Scale bar = 50 μm. (J) Microglia activation rate. Dots in panels represent individual samples. Data are presented as mean ± SEM. *P < 0.05, **P< 0.01, ***P < 0.001. Fig. S4. Correlation analysis and gut microbiota signatures of donor [file 40168_2023_1656_MOESM1_ESM.zip › Supplementary Fig. S8.tif]
