# Supplementary material for: Microbiota-indole 3-propionic acid-brain axis mediates abnormal synaptic pruning of hippocampal microglia and susceptibility to ASD in IUGR offspring
Source: Microbiome. 2023 Nov 7;11:245. doi: 10.1186/s40168-023-01656-1 (PMC10629055; doi:10.1186/s40168-023-01656-1)
Supplement: Supplementary file 3 — Additional file 2: Table S1. |List of donors. Table S2. |Sequences of primers used for RT-qPCR. [file 40168_2023_1656_MOESM2_ESM.docx]

**Supplementary Table 1 |List of donors.**

| **Visit number** | **Group** | **Sex** | **GA at birth (wks)** | **Age at IPA test (d)** | **Age at IPA test (wks)** | **Corrected age (wks)** | **Body weight (kg)** | **Pregnancy mode** |
| --- | --- | --- | --- | --- | --- | --- | --- | --- |
| 1 | Control | M | 38 | 15 | 2.1 | 1.1 | 3.6 | Caesarean birth |
| 2 | Control | M | 40 | 19 | 2.7 | 3.7 | 3.0 | Caesarean birth |
| 3 | Control | M | 39 | 43 | 6.1 | 6.1 | 3.1 | Caesarean birth |
| 4 | Control | M | 41 | 48 | 6.9 | 8.9 | 2.6 | Caesarean birth |
| 5 | Control | M | 39 | 62 | 8.9 | 8.9 | 2.7 | Caesarean birth |
| 6 | Control | M | 38 | 90 | 12.9 | 11.9 | 3.0 | Caesarean birth |
| 7 | Control | M | 38 | 111 | 15.9 | 14.9 | 3.2 | Caesarean birth |
| 8 | Control | M | 39 | 170 | 24.3 | 24.3 | 3.1 | Caesarean birth |
| 9 | Control | M | 39 | 228 | 32.6 | 32.6 | 2.9 | Caesarean birth |
| 10 | Control | M | 39 | 186 | 26.6 | 26.6 | 2.8 | Caesarean birth |
| 11 | Control | M | 39 | 180 | 25.7 | 25.7 | 2.8 | Caesarean birth |
| 12 | Control | M | 37 | 132 | 18.9 | 16.9 | 2.8 | Caesarean birth |
| 13 | Control | M | 41 | 214 | 30.6 | 32.6 | 3.6 | Caesarean birth |
| 14 | Control | M | 38 | 234 | 33.4 | 32.4 | 2.8 | Caesarean birth |
| 15 | Control | M | 40 | 270 | 38.6 | 39.6 | 3.3 | Caesarean birth |
| 16 | Control | M | 39 | 365 | 52.1 | 52.1 | 2.8 | Caesarean birth |
| 17 | Control | M | 37 | 150 | 21.4 | 19.4 | 3.3 | Caesarean birth |
| 18 | Control | M | 37 | 189 | 27.0 | 25.0 | 3.2 | Caesarean birth |
| 19 | Control | M | 38 | 195 | 27.9 | 26.9 | 3.1 | Caesarean birth |
| 20 | Control | M | 38 | 202 | 28.9 | 27.9 | 3.1 | Caesarean birth |
| 1 | IUGR | M | 34 | 2 | 0.3 | -4.7 | 1.7 | Caesarean birth |
| 2 | IUGR | M | 34 | 2 | 0.3 | -4.7 | 2.0 | Caesarean birth |
| 3 | IUGR | M | 31 | 15 | 2.1 | -5.9 | 1.8 | Caesarean birth |
| 4 | IUGR | M | 32 | 18 | 2.6 | -4.4 | 2.1 | Caesarean birth |
| 5 | IUGR | M | 34 | 49 | 7.0 | 2.0 | 2.1 | Caesarean birth |
| 6 | IUGR | M | 31 | 65 | 9.3 | 1.3 | 2.0 | Caesarean birth |
| 7 | IUGR | M | 35 | 83 | 11.9 | 7.9 | 2.4 | Caesarean birth |
| 8 | IUGR | M | 34 | 112 | 16.0 | 11.0 | 2.5 | Caesarean birth |
| 9 | IUGR | M | 35 | 115 | 16.4 | 12.4 | 2.2 | Caesarean birth |
| 10 | IUGR | M | 28 | 115 | 16.4 | 5.4 | 2.2 | Caesarean birth |
| 11 | IUGR | M | 32 | 120 | 17.1 | 10.1 | 2.1 | Caesarean birth |
| 12 | IUGR | M | 35 | 125 | 17.9 | 13.9 | 2.2 | Caesarean birth |
| 13 | IUGR | M | 33 | 0 | 0.0 | -6.0 | 1.5 | Caesarean birth |
| 14 | IUGR | M | 34 | 185 | 26.4 | 21.4 | 2.4 | Caesarean birth |
| 15 | IUGR | M | 35 | 183 | 26.1 | 22.1 | 2.1 | Caesarean birth |
| 16 | IUGR | M | 31 | 243 | 34.7 | 26.7 | 1.9 | Caesarean birth |
| 17 | IUGR | M | 33 | 262 | 37.4 | 31.4 | 2.4 | Caesarean birth |
| 18 | IUGR | M | 31 | 262 | 37.4 | 29.4 | 2.0 | Caesarean birth |
| 19 | IUGR | M | 31 | 277 | 39.6 | 31.6 | 2.5 | Caesarean birth |
| 20 | IUGR | M | 31 | 292 | 41.7 | 33.7 | 1.8 | Caesarean birth |
| 21 | IUGR | M | 32 | 36 | 5.1 | -1.9 | 2.1 | Caesarean birth |

**Supplementary Table 2 |Sequences of primers used for RT-qPCR.**

| Target | Forward Primer (5’–3’) | Reverse Primer (5’–3’) |
| --- | --- | --- |
| *Psd95* | ACCAGAAGAGTATAGCCGATTCG | GGTCTTGTCGTAGTCAAACAGG |
| *Syn* | AACAACAGTAGCATCAGCAGCAGAG | CACCAGAAGCAGGAATCATATCTCAGG |
| *Nf-κb* | GATGTCAACAGAGTAACCTACC | GTCATAGCTCTCCTCATCCT |
| *Ahr* | CAGGACGAGTCCATCTATCT | CCTGAGTGTGTCTGATTTCC |
| *Gapdh* | GCCTCCAAGGAGTAAGAAAC | GTCTGGGATGGAATTGTGAG |
